# Korea Seroprevalence Study of Monitoring of SARS-COV-2 Antibody Retention and Transmission (K-SEROSMART): findings from national representative sample

**DOI:** 10.4178/epih.e2023075

**Published:** 2023-08-17

**Authors:** Jina Han, Hye Jin Baek, Eunbi Noh, Kyuhyun Yoon, Jung Ae Kim, Sukhyun Ryu, Kay O Lee, No Yai Park, Eunok Jung, Sangil Kim, Hyukmin Lee, Yoo-Sung Hwang, Jaehun Jung, Hun Jae Lee, Sung-il Cho, Sangcheol Oh, Migyeong Kim, Chang-Mo Oh, Byengchul Yu, Young-Seoub Hong, Keonyeop Kim, Sunjae Jung, Mi Ah Han, Moo-Sik Lee, Jung-Jeung Lee, Young Hwangbo, Hyeon Woo Yim, Yu-Mi Kim, Joongyub Lee, Weon-Young Lee, Jae-Hyun Park, Sungsoo Oh, Heui Sug Jo, Hyeongsu Kim, Gilwon Kang, Hae-Sung Nam, Ju-Hyung Lee, Gyung-Jae Oh, Min-Ho Shin, Soyeon Ryu, Tae-Yoon Hwang, Soon-Woo Park, Sang Kyu Kim, Roma Seol, Ki-Soo Park, Su Young Kim, Jun-wook Kwon, Sung Soon Kim, Byoungguk Kim, June-Woo Lee, Eun Young Jang, Ah-Ra Kim, Jeonghyun Nam, The Korea Community Health Survey Group, Soon Young Lee, Dong-Hyun Kim

**Affiliations:** 1 Department of Preventive Medicine and Public Health, Ajou University School of Medicine, Suwon, Korea; 2 National Radiation Emergency Medical Center, Korea Institute of Radiological and Medical Sciences, Seoul, Korea; 3 Institute of Health and Environment, Seoul National University, Seoul, Korea; 4 Department of Nursing, Kyungmin University, Uijeongbu, Korea; 5 Department of Preventive Medicine, Konyang University College of Medicine, Daejeon, Korea; 6 Gallup Korea, Seoul, Korea; 7 Graduate School of Public Health, Inje University, Seoul, Korea; 8 Department of Mathematics, Konkuk University, Seoul, Korea; 9 Department of Internal Medicine, College of Medicine, The Catholic University, Seoul, Korea; 10 Department of Laboratory Medicine, Yonsei University College of Medicine, Seoul, Korea; 11 Seegene Medical Foundation, Seoul, Korea; 12 Department of Preventive Medicine, Gachon University College of Medicine, Incheon, Korea; 13 Department of Social and Preventive Medicine, Inha University College of Medicine, Incheon, Korea; 14 Department of Public Health Science, Graduate School of Public Health, Seoul National University, Seoul, Korea; 15 MAPO-gu Public Health Center, Seoul, Korea; 16 GUNPO-si Public Health Center, Gunpo, Korea; 17 Department of Preventive Medicine, Kyung Hee University School of Medicine, Seoul, Korea; 18 Department of Preventive Medicine, Kosin University College of Medicine, Busan, Korea; 19 Department of Preventive Medicine, Dong-A University College of Medicine, Busan, Korea; 20 Department of Preventive Medicine, Kyungpook National University School of Medicine, Daegu, Korea; 21 Department of Preventive Medicine, Yonsei University College of Medicine, Seoul, Korea; 22 Department of Preventive Medicine, Chosun University College of Medicine, Gwangju, Korea; 23 Department of Preventive Medicine, Keimyung University School of Medicine, Daegu, Korea; 24 Department of Preventive Medicine, Soonchunhyang University College of Medicine, Cheonan, Korea; 25 Department of Preventive Medicine, College of Medicine, The Catholic University, Seoul, Korea; 26 Department of Preventive Medicine, Hanyang University College of Medicine, Seoul, Korea; 27 School of Public Health, Hanyang University, Seoul, Korea; 28 Department of Preventive Medicine, Seoul National University College of Medicine, Seoul, Korea; 29 Institute of Health Policy and Management, Medical Research Center, Seoul National University, Seoul, Korea; 30 Department of Preventive Medicine, Chung-Ang University College of Medicine, Seoul, Korea; 31 Department of Preventive Medicine, Sungkyunkwan University School of Medicine, Suwon, Korea; 32 Department of Occupational & Environmental Medicine, Yonsei University Wonju College of Medicine, Wonju, Korea; 33 Department of Health Policy and Management, Kangwon National University School of Medicine, Chuncheon, Korea; 34 Department of Preventive Medicine, Konkuk University School of Medicine, Seoul, Korea; 35 Department of Health Information and Management, Chungbuk National University College of Medicine, Cheongju, Korea; 36 Department of Preventive Medicine, Chungnam National University College of Medicine, Daejeon, Korea; 37 Department of Preventive Medicine, Jeonbuk National University Medical School, Jeonju, Korea; 38 Department of Preventive Medicine, Wonkwang University School of Medicine, Iksan, Korea; 39 Department of Preventive Medicine, Chonnam National University Medical School, Hwasun, Korea; 40 Department of Preventive Medicine & Public Health, Yeungnam University College of Medicine, Gyeongsan, Korea; 41 Department of Preventive Medicine, Daegu Catholic University School of Medicine, Gyeongsan, Korea; 42 Department of Preventive Medicine, Dongguk University College of Medicine, Gyeongju, Korea; 43 Department of Preventive Medicine, Inje University College of Medicine, Busan, Korea; 44 Department of Preventive Medicine, Institute of Health Sciences, Gyeongsang National University College of Medicine, Jinju, Korea; 45 Department of Preventive Medicine, Jeju National University School of Medicine, Jeju, Korea; 46 National Institute of Health, Korea Disease Control and Prevention Agency, Cheongju, Korea; 47 Center for Vaccine Research, National Institute of Infectious Diseases, Korea Disease Control and Prevention Agency, Cheongju, Korea; 48 Division of Vaccine Clinical Research, Center for Vaccine Research, National Institute of Infectious Diseases, Cheongju, Korea; 49 Department of Social and Preventive Medicine, Hallym University College of Medicine, Chuncheon, Korea

**Keywords:** COVID-19, Seroepidemiologic studies, Antibody, Community Health Survey, Sampling studies

## Abstract

**OBJECTIVES:**

We estimated the population prevalence of antibodies to severe acute respiratory syndrome coronavirus 2 (SARS-CoV-2), including unreported infections, through a Korea Seroprevalence Study of Monitoring of SARS-CoV-2 Antibody Retention and Transmission (K-SEROSMART) in 258 communities throughout Korea.

**METHODS:**

In August 2022, a survey was conducted among 10,000 household members aged 5 years and older, in households selected through two stage probability random sampling. During face-to-face household interviews, participants self-reported their health status, COVID-19 diagnosis and vaccination history, and general characteristics. Subsequently, participants visited a community health center or medical clinic for blood sampling. Blood samples were analyzed for the presence of antibodies to spike proteins (anti-S) and antibodies to nucleocapsid proteins (anti-N) SARS-CoV-2 proteins using an electrochemiluminescence immunoassay. To estimate the population prevalence, the PROC SURVEYMEANS statistical procedure was employed, with weighting to reflect demographic data from July 2022.

**RESULTS:**

In total, 9,945 individuals from 5,041 households were surveyed across 258 communities, representing all basic local governments in Korea. The overall population-adjusted prevalence rates of anti-S and anti-N were 97.6% and 57.1%, respectively. Since the Korea Disease Control and Prevention Agency has reported a cumulative incidence of confirmed cases of 37.8% through July 31, 2022, the proportion of unreported infections among all COVID-19 infection was suggested to be 33.9%.

**CONCLUSIONS:**

The K-SEROSMART represents the first nationwide, community-based seroepidemiologic survey of COVID-19, confirming that most individuals possess antibodies to SARS-CoV-2 and that a significant number of unreported cases existed. Furthermore, this study lays the foundation for a surveillance system to continuously monitor transmission at the community level and the response to COVID-19.

## GRAPHICAL ABSTRACT

[Fig f5-epih-45-e2023075]

## INTRODUCTION

**Figure 1.**
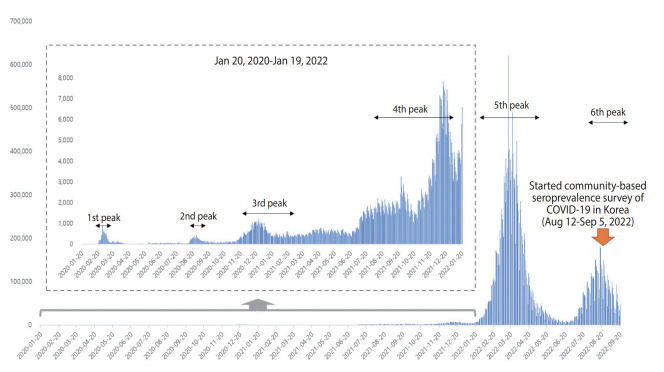
Epidemic curve of coronavirus disease 2019 (COVID-19) in Korea from January 20, 2020 to September 30, 2022.

On March 11, 2020, the World Health Organization declared coronavirus disease 2019 (COVID-19) a global pandemic [1]. As of July 31, 2022, over 580 million confirmed cases of COVID-19 have been reported worldwide, resulting in approximately 6.42 million deaths. In Korea, community outbreak has persisted since the emergence of COVID-19 (Figure 1), with over 19.77 million confirmed cases and more than 25,000 deaths reported [2].

In Korea, the test-trace-isolate-quarantine (TTIQ) containment strategy has been employed since the beginning of the COVID-19 pandemic in 2020 to promptly identify infected individuals and disrupt transmission routes through rapid testing and extensive follow-up [3]. However, in late 2020, the third peak of COVID-19 occurred. From then onward, the proportion of cases with epidemiologic transmission routes unknown at the time of diagnosis increased, resulting in many unreported cases within the community [4]. These cases have been reported to account for 5% to 80% of individuals infected with severe acute respiratory syndrome coronavirus 2 (SARS-CoV-2) in a systematic review and meta-analysis worldwide [5], who shed the virus at similar levels comparable to symptomatic individuals [6,7]. Although they do not require medical care, these cases contribute to the spread of the disease within the community [8].

The World Health Organization recommends population-based seroepidemiologic surveys to determine the prevalence of antibodies to SARS-CoV-2, including asymptomatic infections in the community [9]. Seroepidemiologic surveys of COVID-19 can be used to monitor and assess the level of immunity to the virus in a population by directly measuring the presence of antibodies resulting from natural infection or vaccination [10-12].

Serotracking data reveals that as of November 2022, a total of 3,924 seroprevalence surveys for COVID-19 have been carried out in 140 countries worldwide [13]. A meta-analysis based on some of these serotracker data, excluding studies with incorrect sample frames and methodological shortcomings, highlights that population-based estimates of COVID-19 far exceed reported cases. The study emphasizes the importance of conducting high-quality and standardized collaborative serosurveillance through random sampling to monitor the COVID-19 pandemic and prepare for potential emerging viruses in the future [14]. The United Kingdom adopted a random sampling approach, surveying 150,000 people every month for 23 months starting from May 2020, during the early phase of the pandemic. The Real-time Assessment of Community Transmission Study produced community-based data on antibody-positive rates repeatedly. This data serves as the foundation for understanding the spread of the pandemic and establishing effective quarantine response strategies [15].

The COVID-19 Serosurveillance Network in Australia routinely calculates the seroprevalence of COVID-19 using residual blood from donors aged 18 years and older [16]. In the United States, the prevalence of anti-nucleocapsid proteins of SARS-CoV-2 was reported every 4 weeks from September 2021 to April 2022 using blood samples collected at healthcare facilities for clinical trials [17]. Additionally, extra data on the seroepidemiology of COVID-19 were collected in the 2021-2022 National Health and Nutrition Examination Survey to overcome the limitations of convenience sampling [18].

In Korea, several seroprevalence surveys for COVID-19 have been conducted; however, most of these studies were small-scale and relied on convenience sampling. Furthermore, these surveys did not provide analysis by region, age, or other socio-demographic factors. The use of residual sample from other survey systems has been limited in calculating a representative prevalence of antibodies to SARS-CoV-2 [4,12,19,20]. The prevalence of antibodies to SARS-CoV-2 may vary based on factors such as the survey population, sampling method, timing and location of the survey, and blood sample analysis technique employed [11].

To accurately estimate the scope of COVID-19 transmission, a nationwide survey of a representative sample of the population is necessary. In Korea, the Community Health Survey (CHS) has been conducted since 2008. This survey, utilizing a regionally representative sampling and survey system, is a collaborative effort between the Korea Disease Control and Prevention Agency (KDCA), 17 provinces, 258 community health centers, and 34 universities [21,22]. The Korea Seroprevalence Study of Monitoring of SARS-CoV-2 Antibody Retention and Transmission (K-SEROSMART) aimed to conduct a national seroepidemiologic survey of COVID-19 using the CHS system to estimate the population prevalence of antibodies to SARS-CoV-2.

## MATERIALS AND METHODS

### System for the community-based seroepidemiologic survey of COVID-19

The Korean Society of Epidemiology, which supervised the survey in collaboration with the National Institute of Health of the KDCA, organized 4 teams: sampling design, field survey, specimen analysis, and data analysis teams. The sampling design team selected and managed the primary sampling units (PSUs) to ensure sample representativeness and calculated population-adjusted weights. The field survey team carried out household surveys and collected blood samples. The specimen analysis team transported blood samples to central lab and performed laboratory tests for SARS-CoV-2 antibodies, while the data analysis team compiled the survey and laboratory test results into a database and performed analyses. Furthermore, a multidisciplinary research committee was established to monitor the survey process and make key decisions.

In this study, the field survey team employed a CHS consortium [22], which included 34 universities, 258 community health centers, and 132 community medical clinics. The participating universities coordinated the survey participants, interviewers, and blood collection sites (community health centers or medical clinics) within each region, while also monitoring the survey process and ensuring quality control. Community health centers and medical centers were responsible for monitoring the status of blood collection in their respective regions and carrying out the blood collection procedures (Supplementary Material 1).

The CHS was initiated by the Korea Centers for Disease Control and Prevention in 2008 under the Community Health Act (Article 4). It is a community-based cross-sectional survey conducted among local residents aged 19 years and over who live within the jurisdictions of community health centers. Each year, approximately 900 individuals from each community and 230,000 in total nationwide were surveyed through face-to-face interviews. Furthermore, health statistics on health behaviors and levels of chronic disease at the regional level were generated. For representative sampling, PSUs were selected using probability proportional systematic sampling stratified by *dong* (neighborhood), *eup* (town), *myeon* (township), and housing type, and sample households were selected through systematic sampling. A consortium of 258 community health centers and 34 universities across the country was formed to standardize the survey procedures and implementation system, as well as to establish a sustainable community survey monitoring system by creating a network within the community. The statistical data generated are used for healthcare planning and to evaluate the performance of health projects in the regions of Korea.

### Sample selection

The sample size was set at 10,000 to ensure the representativeness of the prevalence of antibodies to SARS-CoV-2 [23]. The survey population consisted of residents aged 5 years and older, residing in residential housing (apartments or houses) within each PSU at the time of the survey, and who were eligible for blood sampling.

**Figure 2.**
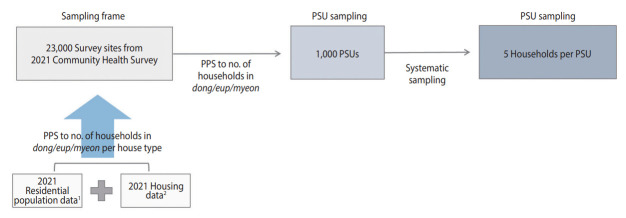
Sampling design process of the Korea Seroprevalence Study of Monitoring of SARS-CoV-2 Antibody Retention and Transmission: August 12-September 5, 2022. SARS-COV-2, severe acute respiratory syndrome coronavirus 2; PPS, probability proportional to size sampling; PSU, primary sampling unit. ^1^Ministry of the Interior and Security in Korea. ^2^Ministry of Land, Infrastructure and Transport in Korea.

The sampling design process is illustrated in Figure 2. The sampling frame for selecting the PSUs was based on 23,000 survey sites from the 2021 CHS, from which 1,000 PSUs were chosen. The PSUs selected from the 2021 CHS were determined using the probability proportional systematic sampling method. This method considered the number of sample points allocated to each *dong*, *eup*, and *myeon*, based on the number of households by housing type in the sampling frame. This frame linked the residential population data from the Ministry of the Interior and Safety with the housing data from the Ministry of Land, Infrastructure, and Transport from 2021 [21,22,24].

The 1,000 PSUs were allocated proportionally, considering the number of population by each *si, gun, gu*, representing all basic local governments. To ensure the participation of all 258 community health centers, 2 PSUs per health center were initially allocated, and the remaining points were distributed based on the population aged 5 years and older in July 2022 for each city, county, and district (Table 1).

In the sampling frame, the number of PSUs allocated to each city, county, and district was selected using the probability proportional systematic sampling method, based on the number of households in *tong* (urban village), *ban* (hamlet), and *ri* (rural village). To account for participant access to blood collection sites, we have excluded PSUs located in isolated areas. In contrast, only 1 health center is in Sejong; however, 10 PSUs were selected to ensure the representativeness of the sample for comparison among provinces.

To choose the sample households for each PSU, we employed the systematic sampling technique. This involved identifying the total number of households within each PSU and selecting 5 primary households along with 2 backup households for each primary household. The reserve households were chosen based on the numbers subsequent to the serial number corresponding to the primary household.

### Survey process

**Figure 3.**
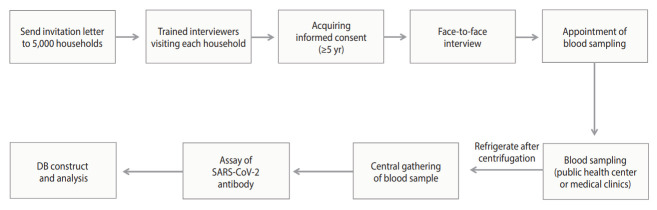
Process of the community-based seroepidemiologic survey of coronavirus disease 2019, Korea Seroprevalence Study of Monitoring of SARS-CoV-2 Antibody Retention and Transmission: August 12-September 5, 2022. SARS-COV-2, severe acute respiratory syndrome coronavirus 2; DB, database.

The survey process is illustrated in Figure 3. Households chosen for the survey received a letter informing them of their eligibility for the study. Trained interviewers visited these households to obtain consent from household members aged 5 years and older. For individuals under the age of 18 years, consent was obtained from a legal representative. The survey was conducted through face-to-face interviews, during which participants self-reported information about their health status, disease history, COVID-19 diagnosis and vaccination history, and socioeconomic status (education, household income, and generational household) to the interviewer. After completing the interview, participants followed the interviewer’s instructions to schedule a time and location for a blood draw.

Blood samples were collected at the community health centers located in the participants’ residential areas, with children aged 5 years to 18 years advised to have their samples collected at designated medical clinics. In accordance with the blood collection protocol, 4 mL of blood was collected from adults and 2 mL from children. The samples were then centrifuged for 10 minutes at 3,000 rpm and subsequently refrigerated.

To ensure the representativeness of the sample, a minimum of 3 households or 10 individuals were surveyed per PSU. Households that either refused to participate or were ineligible for the survey (e.g., those composed of non-resident adults, foreign residents, household members with COVID-19-related symptoms or under quarantine, and those unable to travel for blood collection) were replaced with a designated reserve household. Furthermore, if the target number of participants for each area was not met, additional visits were made to reserve households.

### Laboratory testing

Blood samples were gathered from the community health centers and designated medical clinics, then brought to regional laboratory centers on the same day. These samples were stored according to provided guidelines and transported to the central laboratory for analysis the following morning. Antibody testing for SARS-CoV-2 was conducted using the Elecsys Anti-SARS-CoV-2 reagent (Roche, Mannheim, Germany) in accordance with the manufacturer’s protocol. The testing was performed on a Cobas e801 analyzer (Roche), utilizing the electrochemiluminescence immunoassay principle. The clinical sensitivity and specificity of this test were 99.5% and 99.8%, respectively [4,25].

Spike proteins and nucleocapsid proteins serve as the primary antigens for detecting antibodies to SARS-CoV-2. The spike protein enables the virus to interact with human cells, while the nucleocapsid protein, as the most abundant protein in the coronavirus, is believed to play a role in viral particle assembly and release [26]. Antibodies to spike proteins (anti-S) and antibodies to nucleocapsid proteins (anti-N) are utilized as indicators of an effective immune response including vaccine effectiveness and as biomarkers of natural infection, respectively [27].

The results of the anti-S assay were presented numerically and deemed positive for anti-S when the cut-off index was greater than or equal to 0.80 U/mL, signifying the formation of antibodies due to natural infection or vaccination [28]. The results of the anti-N assay were classified as reactive or non-reactive, with a specified cut-off index. A cut-off index of 1.0 or higher indicated an anti-N result of reactive, meaning that the patient tested positive for anti-N; this suggested that antibodies had previously formed due to natural infection [28]. Importantly, the degree to which the cut-off index for the anti-N assay is higher than 1.0 does not represent the total quantity of antibodies present in the sample.

### Statistical analysis

To estimate the population prevalence of anti-S and anti-N, we employed the PROC SURVEYMEANS procedure in SAS version 9.4 (SAS Institute Inc., Cary, NC, USA), utilizing sample weights based on the July 2022 registered population [22-24].

The sample weight was calculated by multiplying 3 factors: (1) sampling design weight, (2) non-response correction weight, and (3) post-stratification correction weight. (i) Sampling design weight is the reciprocal of the sampling rate, which was determined by dividing the number of people assigned to a health center unit by the number of registered residents. (ii) Non-response correction weight was calculated as the inverse of the response rate by health center unit, under the assumption that the characteristics of non-respondents and respondents were similar. (iii) Post-stratification adjustment weights were used to equalize the distribution of the sample by sex and age group, both of which influence antibody prevalence, with the distribution of the population. These weights were calculated by dividing the number of residents by sex and age group at each site by the sum of the design weight and the non-response adjustment weight.

The prevalence of anti-S and anti-N is presented with 95% confidence intervals (CIs) and relative standard error (RSE). The RSE was calculated by dividing the standard error by the corresponding estimate and expressing it as a percentage (%). In household surveys, an RSE of 25% or lower is considered to indicate high confidence in the estimated value [29].

In this study, the difference between the anti-N prevalence and the cumulative COVID-19 incidence reported by the KDCA was defined as the unreported infection rate [4]. To compare the differences in cumulative incidence by sex, age, and provinces, the anti-N prevalence was further calculated using age-standardized weighting, with the December 2021 population serving as the reference.

The cumulative incidence of COVID-19 was determined using the total number of confirmed cases reported to the Korean COVID-19 case reporting system between January 20, 2020 and July 31, 2022 as the numerator, and the resident population in December 2021 as the denominator.

The reporting system for confirmed COVID-19 cases in Korea operates as follows. Medical institutions are required to report a confirmed case (either a positive professional rapid antigen test or an emergency screening test result) to the community health center within 24 hours of initially identifying the case, then enter the case report into the COVID-19 Information Management System. Community health centers must then report the case to the city health department within 24 hours of initially identifying the case (upon confirmation of a positive result from a health and environmental laboratory or testing contractor) and enter the outbreak report into the COVID-19 Information Management System [30].

### Ethics statement

This survey received an exemption from review by the Institutional Review Board of the KDCA, in accordance with Article 36 of the Bioethics Act, Article 33 of the Enforcement Rules, and Article 2 of the Bioethics Act. The study was deemed to require urgent action for public health and was conducted directly or commissioned by the state or local government to review and evaluate public welfare or service programs (2022-07-04-PE-A).

## RESULTS

The face-to-face interviews took place from August 11, 2022 to August 30, 2022, while blood samples were collected between August 12, 2022 and September 5, 2022. In total, 9,945 individuals from 5,041 households participated in the survey. These participants included 4,008 individuals from 1,957 primary households and 5,938 individuals from 3,084 backup households. The prevalence of anti-S and anti-N, based on various characteristics, is presented in Table 2.

**Figure 4.**
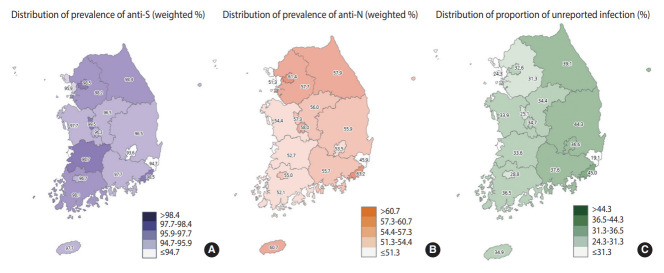
Distribution of severe acute respiratory syndrome coronavirus 2 (SARS-CoV-2) antibody prevalence (A: anti-S, B: anti-N) and proportion of unreported infections (C) by region of the Korea Seroprevalence Study of Monitoring of SARS-CoV-2 Antibody Retention and Transmission: August 12-September 5, 2022. anti-S, antibodies to spike proteins; anti-N, antibodies to nucleocapsid proteins.

The prevalence of anti-S was 97.6% (95% CI, 97.2 to 97.9). By age group, the anti-S prevalence was lowest among 5-9-year-olds at 81.5% (95% CI, 76.5 to 86.4) and increased with age, reaching a peak of 99.4% (95% CI, 98.9 to 99.8) in the 70-79-year group. Significant differences were also found based on household income and generational household. In terms of provinces, the prevalence of anti-S proteins was lowest in Daegu at 93.6% (95% CI, 90.8 to 96.3) and highest in Sejong at 99.5% (95% CI, 98.4 to 100.0), with statistically significant differences observed between provinces (Figure 4A).

The prevalence of anti-N was found to be 57.1% (95% CI, 56.0 to 58.2), with rates of 56.4% (95% CI, 54.7 to 58.1) in male and 57.9% (95% CI, 56.4 to 59.3) in female. By age group, the highest prevalence of anti-N proteins was observed in 5-9-year-olds at 82.1% (95% CI, 77.3 to 87.0), and it decreased with age, reaching its lowest point of 31.2% (95% CI, 27.2 to 35.2) in individuals aged 80 years and older. The anti-N prevalence increased with education and household income, with the lowest prevalence of 44.6% (95% CI, 41.3 to 47.9) found in single-person households and the highest prevalence of 60.8% (95% CI, 59.2 to 62.4) in second generation. By province, the lowest prevalence of anti-N was found in Ulsan at 45.9% (95% CI, 37.7 to 54.1) and the highest in Busan at 63.2% (95% CI, 59.3 to 67.2), with statistically significant differences between provinces (Figure 4B).

The proportion of unreported infections was 33.9% overall (36.6% in male and 30.5% in female participants). Regarding age group, the highest proportions were observed in those 50-59 years, 60-69 years, and 70-79 years old, at 47.9%, 45.0%, and 40.5%, respectively, while the lowest proportion was found in those 80 years and older, at 12.9% (Table 3). By metropolitan area, the lowest proportion in Ulsan (19.1%) and the highest in Busan (45.0%), constituting a 2.4-fold difference in the proportion of unreported infections between the regions (Figure 4C).

## DISCUSSION

In this study, we carried out a seroepidemiologic survey of COVID-19 among 9,945 residents aged 5 years and older from August 11, 2022 to September 5, 2022, several months after the surge of COVID-19 cases mainly due to Omicron variants in early 2022 in Korea. The K-SEROSMART was based on the CHS system and was designed to determine the representative population prevalence of anti-S and anti-N in Korea.

The prevalence of anti-S was 97.6%, indicating that most Koreans possess anti-S. No significant differences were observed based on sex or region; however, the prevalence of anti-S was lower among 5-9-year-olds (81.5%) compared to other age groups. As of August 2022 in Korea, despite recommendations of at least 1 dose for children aged 12-17 years and high-risk children aged 5-11 years [31], the vaccination rate for 5-11-year-olds was only 1.5% [32]. Furthermore, the prevalence of anti-N among 5-9-year-olds was 82.1%, similar to the prevalence of anti-S proteins, suggesting that the anti-S produced in this age group is probably due to natural infection.

The anti-N prevalence rate was 57.1%. No significant differences were observed by sex; however, the prevalence of anti-N proteins decreased with increasing age. In another study, the prevalence of anti-N, as estimated from 5,005 blood samples collected between August 23, 2022 and September 2, 2022, in Australia, was 65.2% [16], Additionally, the prevalence from 13,787 blood samples collected between June 29, 2022 and July 19, 2022, in the United Kingdom was 73.4% [33]. This could be interpreted either as a success in preventing the spread of COVID-19 in Korea or as an indication of the potential for COVID-19 to spread [34].

Since the onset of the COVID-19 outbreak, Korea has implemented a swift and comprehensive response system, involving numerous organizations alongside the TTIQ strategy. Furthermore, its citizens have actively participated in preventative measures such as wearing masks, practicing social distancing, and becoming vaccinated. Consequently, the scale of COVID-19 infections in Korea has been much smaller than in other countries.

Evidence indicates that COVID-19 vaccination reduces the incidence, severity, hospitalization, and mortality of COVID-19 [35]. In Korea, individuals aged 50 years and older have been prioritized for COVID-19 vaccination. Consequently, as of July 2022, the vaccination rate for this age group reached 96.7%, and the booster update rate was 87.0%, with vaccination rates increasing with age [36]. This finding suggests that the prevalence of anti-N positivity decreases with age, possibly indicating the effectiveness of the vaccine. However, the lower anti-N prevalence in older age groups and among those with comorbidities implies that they may still be at risk of natural infection. These individuals could potentially become a high-risk patient population in the event of an outbreak and should be considered for ongoing monitoring and response measures.

In Korea, the proportion of unreported infections was 33.9%. This represents a significant increase from the 18.3% [37] of unreported infections reported in the residual blood testing of participants in the National Health and Nutrition Examination Survey in April 2022. The likely cause of this increase is the rise in the number of unreported infections due to the Omicron pandemic. However, this figure is lower than the 43.7% of unreported infections among adults aged 18 years and older reported in the 2021-2022 National Health and Nutrition Examination Survey in the United States [18]. This difference may be attributed to the extensive follow-up of patient contacts under the TTIQ policy in the early stages of the COVID-19 outbreak, as previously described [3].

Following the Omicron surge in early 2022, the TTIQ policy was relaxed, which may have further increased the proportion of hidden COVID-19 cases in the community. The proportion of unreported infections is an important measure of the extent of COVID-19 transmission within a community [38-40]. In the present study, the proportion of unreported infections was higher among male participants and those aged 50-59 years and 60-69 years, and it varied by up to a factor of approximately 2.4 across provinces. The impact of the COVID-19 pandemic was disproportionately influenced by individual socioeconomic characteristics, and studies have reported differences in response capacity and policy compliance [41,42]. Further investigation and in-depth analysis of socioeconomic factors such as occupation and employment type that may influence differences in the proportion of unreported infections, as well as COVID-19 policies in each region, are needed to develop complementary policies that consider socioeconomic characteristics.

By utilizing an existing consortium of community health centers and universities [21] and forming a collaborative network with regional medical clinics, the present survey efficiently constituted a systematic, rapid, and cost-effective seroepidemiologic investigation of COVID-19. This will provide a foundation for future monitoring of COVID-19 antibodies by tracking existing participants to observe changes in antibody levels. Additionally, it will persist in facilitating observation of the spread of and response to COVID-19 within the community, while also functioning as a surveillance system for other emerging infectious diseases.

Limitations of this study include the following. First, the prevalence of COVID-19 antibodies may be underestimated in some or all populations. This community-based cross-sectional survey may be subject to survivorship bias, as deaths or certain hospitalizations due to COVID-19 (particularly common among older adults) would have prevented inclusion. Furthermore, even in cases of natural infection, antibody formation may not have occurred or antibody titers could have been lost over time; this depends on factors such as the level of pathogen exposure, individual susceptibility to disease, timing of vaccination and natural infection, underlying medical conditions, and the presence of breakthrough infections [17,43-45]. Second, the participation rate of children and young people, who had difficulty in visiting community health centers or medical clinics for blood sampling, was low. However, the parameters were estimated by weighting under the assumption that no difference existed in antibody prevalence based on survey participation. Third, we used the cumulative number of COVID-19 cases reported by the KDCA to estimate the scale of unreported infections in the community. Since the diagnosis of COVID-19 was only available for patients who visited a medical institution or health center, the rate of unreported infection in the community may be underestimated.

In August 2022, a nationwide community-based seroepidemiologic survey for COVID-19 revealed that most of the Korean population had acquired anti-S through vaccination or natural infection, and that 1 in 3 naturally infected individuals had an unreported infection. This survey is the first of its kind in Korea to determine a representative COVID-19 antibody prevalence rate by systematically and cost-effectively surveying many people within a short period (about 3 weeks). This was achieved through collaboration with community health centers, universities, and medical clinics in the region. The survey is also noteworthy for establishing the foundation of a surveillance system that can be used to continuously monitor the spread of COVID-19 infection and regional responses. In addition to follow-up and periodic surveys of the participants, the data will be used as a basis for developing COVID-19 prevention policies. This will be accomplished by identifying the characteristics of COVID-19 spread in the community, factors influencing undetected infections, and fluctuations in antibody titers through linkage with various sources. These sources include COVID-19 confirmation information and vaccination history from the KDCA, as well as morbidity information from the Korea Health Insurance Corporation.

## Figures and Tables

**Figure f5-epih-45-e2023075:**
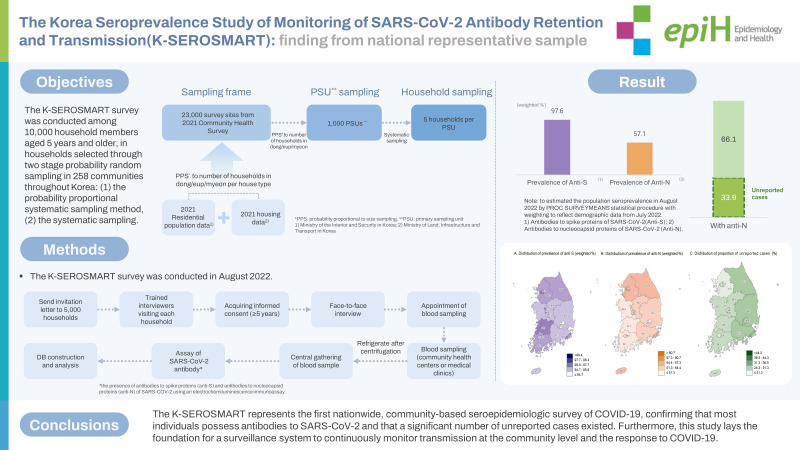


**Table 1.** Distribution of PSUs by region in the Korea Seroprevalence Study of Monitoring of SARS-CoV-2 Antibody Retention and Transmission

Province	Community health centers (n)	Population	Priority allocation (PSU)	Proration (PSU)	Total (PSU)
Seoul	25	9,493,211	50	89	139
Busan	16	3,334,595	32	31	63
Daegu	8	2,374,120	16	22	38
Incheon	10	2,960,580	20	28	48
Gwangju	5	1,434,703	10	13	23
Daejeon	5	1,448,182	10	13	23
Ulsan	5	1,114,753	10	10	20
Sejong	1	380,889	2	8	10
Gyeonggi	48	13,589,362	96	126	222
Gangwon	18	1,539,178	36	14	50
Chungbuk	14	1,597,163	28	15	43
Chungnam	16	2,121,011	32	20	52
Jeonbuk	14	1,776,949	28	17	45
Jeonnam	22	1,825,534	44	17	61
Gyeongbuk	25	2,612,016	50	24	74
Gyeongnam	20	3,293,709	40	31	71
Jeju	6	678,491	12	6	18
Total	258	51,574,446	516	484	1,000

PSU, primary sampling unit; SARS-COV-2, severe acute respiratory syndrome coronavirus 2.

**Table 2.** Prevalence of antibodies to SARS-CoV-2 based on socio-demographic characteristics of the Korea Seroprevalence Study of Monitoring of SARS-CoV-2 Antibody Retention and Transmission: August 12-September 5, 2022

Characteristics		n	Prevalence of anti-S			Prevalence of anti-N		
			Weighted % (95% CI)	RSE	p-value^1^	Weighted % (95% CI)	RSE	p-value^1^
Overall		9,945	97.6 (97.2, 97.9)	0.2	-	57.1 (56.0, 58.2)	1.0	-
Sex	Male	4,474	97.2 (96.6, 97.7)	0.3	0.024	56.4 (54.7, 58.1)	1.5	0.187
	Female	5,471	98.0 (97.5, 98.4)	0.2		57.9 (56.4, 59.3)	1.3	
Age (yr)	5-9	297	81.5 (76.5, 86.4)	3.1	<0.001	82.1 (77.3, 87.0)	3.0	<0.001
	10-19	757	92.9 (90.9, 94.8)	1.1		69.3 (65.8, 72.8)	2.6	
	20-29	887	98.8 (98.1, 99.6)	0.4		60.7 (57.1, 64.2)	3.0	
	30-39	934	98.4 (97.5, 99.3)	0.5		61.5 (58.1, 64.9)	2.8	
	40-49	1,337	98.6 (97.9, 99.3)	0.4		59.7 (56.8, 62.6)	2.5	
	50-59	1,660	99.2 (98.7, 99.7)	0.2		54.5 (51.9, 57.2)	2.5	
	60-69	1,994	99.2 (98.8, 99.7)	0.2		50.4 (47.9, 52.8)	2.5	
	70-79	1,450	99.4 (98.9, 99.8)	0.2		43.0 (40.2, 45.8)	3.3	
	≥80	629	97.3 (95.8, 98.8)	0.8		31.2 (27.2, 35.2)	6.5	
Education (age ≥19 yr)	Primary school	1,555	99.1 (98.6, 99.6)	0.3	0.721	40.1 (37.4, 42.9)	3.4	<0.001
	Middle/High school	3,702	98.9 (98.5, 99.3)	0.2		54.4 (52.5, 56.2)	1.7	
	Postsecondary	3,582	98.7 (98.3, 99.2)	0.2		58.2 (56.4, 60.0)	1.6	
Household income (/103 KRW)	<2,000	1,940	97.8 (97.0, 98.7)	0.5	0.007	43.1 (40.5, 45.7)	3.1	<0.001
	2,000-3,999	2,391	98.3 (97.7, 98.9)	0.3		56.7 (54.4, 59.0)	2.1	
	4,000-5,999	1,369	96.4 (95.3, 97.5)	0.6		59.0 (56.1, 62.0)	2.5	
	6,000-7,999	1,565	96.7 (95.6, 97.7)	0.5		57.4 (54.7, 60.2)	2.5	
	≥8,000	1,288	97.9 (97.0, 98.8)	0.5		64.7 (61.9, 67.6)	2.3	
Generational household	Single person	1,234	98.4 (97.5, 99.3)	0.5	<0.001	44.6 (41.3, 47.9)	3.8	<0.001
	First generation	2,802	99.3 (99.0, 99.7)	0.2		52.3 (50.1, 54.4)	2.1	
	Second generation	4,195	96.4 (95.8, 97.1)	0.3		60.8 (59.2, 62.4)	1.4	
	Third generation	526	97.6 (96.0, 99.2)	0.9		58.4 (53.6, 63.2)	4.2	
Province	Seoul	1,399	98.5 (97.8, 99.2)	0.3	<0.001	61.4 (58.7, 64.2)	2.3	<0.001
	Busan	636	98.5 (97.6, 99.4)	0.5		63.2 (59.3, 67.2)	3.2	
	Daegu	382	93.6 (90.8, 96.3)	1.5		53.5 (48.2, 58.8)	5.1	
	Incheon	485	95.9 (94.1, 97.8)	1.0		51.3 (46.0, 56.6)	5.3	
	Gwangju	228	96.7 (94.2, 99.3)	1.3		55.0 (47.8, 62.2)	6.7	
	Daejeon	230	96.3 (93.7, 98.9)	1.4		58.0 (50.9, 65.1)	6.3	
	Ulsan	192	94.7 (91.0, 98.3)	2.0		45.9 (37.7, 54.1)	9.1	
	Sejong	100	99.5 (98.4, 100.0)	0.5		57.3 (47.4, 67.1)	8.8	
	Gyeonggi	2,173	98.2 (97.6, 98.8)	0.3		57.7 (55.5, 59.9)	2.0	
	Gangwon	495	98.4 (97.1, 99.8)	0.7		57.9 (53.3, 62.6)	4.1	
	Chungbuk	431	96.5 (94.4, 98.6)	1.1		56.0 (50.4, 61.6)	5.1	
	Chungnam	513	97.7 (96.0, 99.4)	0.9		54.4 (49.3, 59.5)	4.8	
	Jeonbuk	440	98.7 (97.3, 100.0)	0.7		52.7 (47.0, 58.5)	5.5	
	Jeonnam	611	98.1 (96.7, 99.4)	0.7		52.1 (47.8, 56.4)	4.2	
	Gyeongbuk	738	96.5 (94.8, 98.2)	0.9		55.9 (51.8, 59.9)	3.7	
	Gyeongnam	710	97.7 (96.2, 99.1)	0.8		55.7 (51.4, 60.1)	4.0	
	Jeju	182	97.7 (94.4, 100.0)	1.7		60.7 (53.1, 68.4)	6.4	

Seroprevalence was estimated with sampling weights using the population of registered residents in July 2022. SARS-CoV-2, severe acute respiratory syndrome coronavirus 2; CI, confidence interval; RSE, relative standard error; anti-S, antibodies to spike proteins; anti-N, antibodies to nucleocapsid proteins; KRW, Korean won.

1 From the Rao-Scott chi-square test.

**Table 3.** Proportion of unreported infections by sex, age group, and region of the Korea Seroprevalence Study of Monitoring of SARS-COV-2 Antibody Retention and Transmission: August 12-September 5, 2022

Characteristics		Age-standardized prevalence rate of anti-N^1^ (A)	Cumulative rate of confirmed COVID-19 reported by KCDC^2^ (B)	Proportion of unreported infections ([A−B]/A)
Overall		57.2	37.8	33.9
Sex				
	Male	56.5	35.8	36.6
	Female	58.0	40.3	30.5
Age (yr)				
	5-9	82.2	66.6	19.0
	10-19	69.5	55.3	20.4
	20-29	60.3	44.2	26.7
	30-39	61.7	43.4	29.7
	40-49	59.7	37.3	37.5
	50-59	54.5	28.4	47.9
	60-69	50.4	27.7	45.0
	70-79	42.7	25.4	40.5
	≥80	30.9	26.9	12.9
Province				
	Seoul	61.6	41.5	32.6
	Busan	63.6	35.0	45.0
	Daegu	53.5	33.9	36.6
	Incheon	51.1	38.7	24.3
	Gwangju	54.6	38.9	28.8
	Daejeon	57.9	37.8	34.7
	Ulsan	46.1	37.3	19.1
	Sejong	53.7	40.2	25.1
	Gyeonggi	56.9	39.1	31.3
	Gangwon	59.6	36.3	39.1
	Chungbuk	56.7	37.2	34.4
	Chungnam	54.8	36.2	33.9
	Jeonbuk	53.9	35.8	33.6
	Jeonnam	54.0	34.3	36.5
	Gyeongbuk	57.5	32.0	44.3
	Gyeongnam	56.1	35.0	37.6
	Jeju	60.2	39.2	34.9

Values are presented as %. KCDC, Korea Centers for Disease Control and Prevention; COVID-19, coronavirus disease 2019; anti-N, antibodies to nucleocapsid proteins; SARS-COV-2, severe acute respiratory syndrome coronavirus 2.

1 Estimated with age-standardized weights using the population of registered residents in December 2021.

2 The cumulative incidence rate was calculated by using the cumulative number of people confirmed by KCDC to have COVID-19 through July 31, 2022 as the numerator and the registered resident population in December 2021 as the denominator.

## SUPPLEMENTARY MATERIALS

Korean version is available at https://www.e-epih.org/.

Supplement Material 1. System for the K-SEROSMART.
