# Supplementary material for: Korea Seroprevalence Study of Monitoring of SARS-COV-2 Antibody Retention and Transmission (K-SEROSMART): findings from national representative sample
Source: Epidemiol Health. 2023 Aug 17;45:e2023075. doi: 10.4178/epih.e2023075 (PMC10728614; doi:10.4178/epih.e2023075)
Supplement: Supplement Material 1. — System for the K-SEROSMART. [file epih-45-e2023075-Supplementary.pptx]

## Slide 1
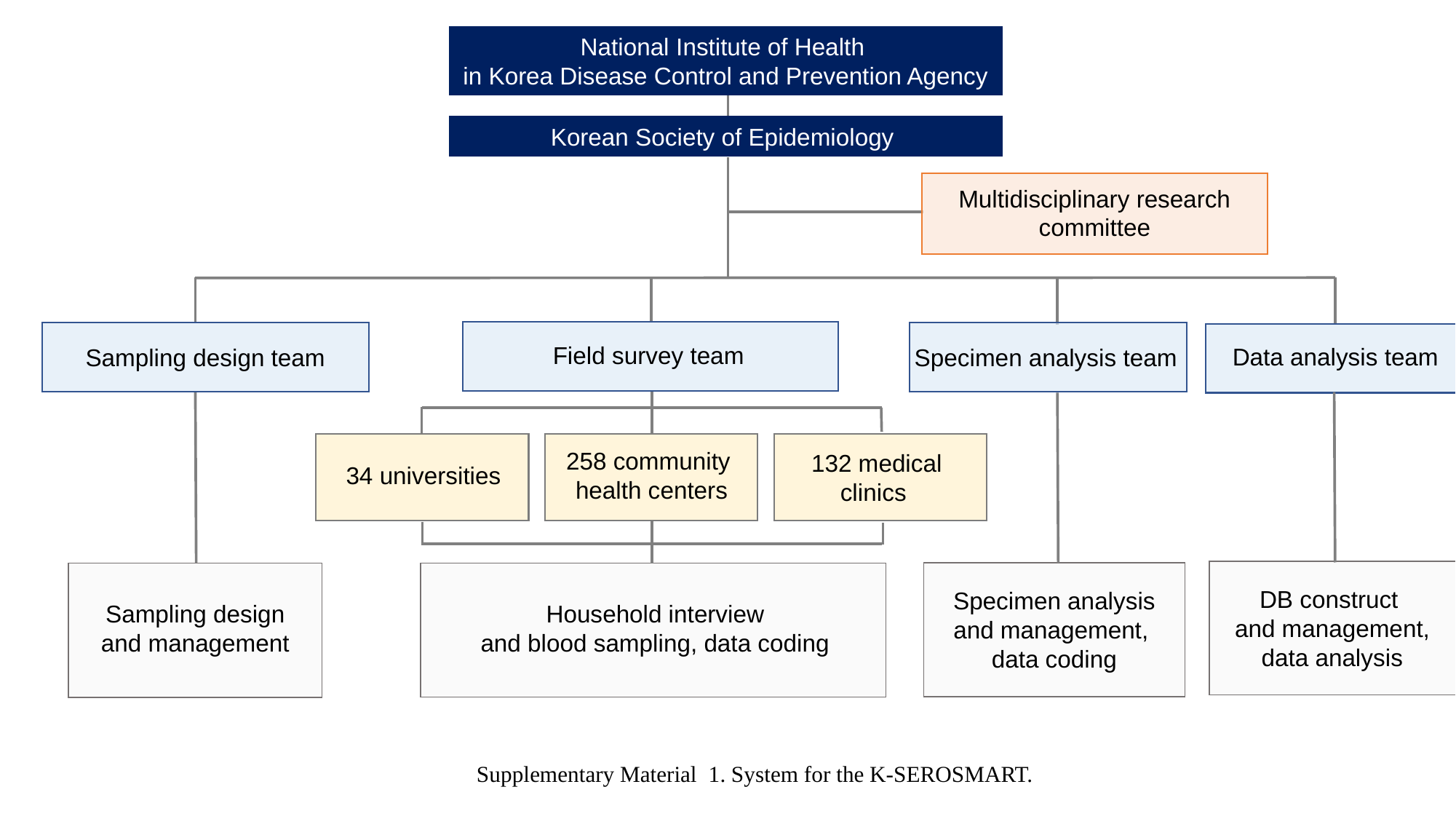

National Institute of Health
in Korea Disease Control and Prevention Agency
Korean Society of Epidemiology
Multidisciplinary research committee
Field survey team
Data analysis team
Specimen analysis team
Sampling design team
258 community health centers
132 medical clinics
34 universities
DB construct
and management, data analysis
Specimen analysis and management,
data coding
Sampling design
and management
Household interview
and blood sampling, data coding
Supplementary Material 1. System for the K-SEROSMART.
