# Supplementary material for: Korea Seroprevalence Study of Monitoring of SARS-COV-2 Antibody Retention and Transmission (K-SEROSMART): findings from national representative sample
Source: Epidemiol Health. 2023 Aug 17;45:e2023075. doi: 10.4178/epih.e2023075 (PMC10728614; doi:10.4178/epih.e2023075)
Supplement: Supplementary file 2 [file epih-45-e2023075-Korean-Supplementary.docx]

**Korea Seroprevalence Study of Monitoring of SARS-COV-2 Antibody Retention and Transmission (K-SEROSMART)**

**Objectives:** 전국 258개 시∙군∙구를 기반으로 하는 한국의 COVID-19 항체유병률 조사(K-SEROSMART)를 수행함으로써 미확진 감염자를 포함한 대표성 있는 COVID-19의 항체유병률을 산출하고자 하였다.

**Methods**: 본 조사는 확률비례계통추출법 및 계통추출법으로 선정된 가구에 거주하는 만 5세 이상 가구원 10,000명을 대상으로 2022년 8월에 수행되었다. 가구방문 면접조사를 통해 연구참여에 동의한 대상자들은 건강상태, 기저질환, COVID-19 확진력, 백신접종력 및 기타 사회경제적 요인에 대해 조사되었고 이후 대상자는 거주지의 보건소 또는 지정의료기관에 방문하여 혈액 검체를 제공하였다. 이를 통해 수집된 혈액 검체는 전기화학발광 면역법으로 S항체와 N항체에 대해 분석되었다. COVID-19 항체유병률의 모수를 추정하기 위해 2022년 7월 인구구조를 반영한 가중치를 적용한 PROC SURVEYMEANS 통계프로시져를 사용하였다.

**Results**: 전국적으로 5,041가구에서 9,945명이 조사에 참여하였다. S항체 유병률은 97.6% (95% CI: 97.2-97.9)이었고 N항체 유병률은 57.1% (95% CI: 56.0-58.2)이었다. 2020년 1월 20일부터 2022년 7월 31일까지 질병관리청에서 보고된 누적확진자 발생률은 37.8%로 N항체의 연령표준화된 유병률 57.2%와 비교해볼 때, 미확진 감염분율은 33.9%이었다.

**Conclusion:** 본 조사(K-SEROSMART)는 지역사회를 기반으로 하는 COVID-19 항체유병률 조사를 통해 국민 대부분이 COVID-19에 대한 항체를 가지고 있으며, 이와 더불어 지역사회 내 미확진 감염자도 상당수 존재하는 것을 확인한 국내 최초의 전국단위 조사이다. 이를 통해 지역사회 내에서 COVID-19의 감염확산과 대응을 지속적으로 모니터링 할 수 있는 감시체계의 기반을 마련하였다.

**Keywords**

COVID-19, seroepidemiologic studies, Community Health Survey, Probability sample

**Introduction**

COVID-19는 2020년 3월 11일 세계보건기구에 의해 세계적인 유행병으로 선언되었다[1]. 2022년 7월 31일 기준, 전 세계적으로 약 642만명의 사망자를 포함하여 5억 8천만건 이상의 COVID-19 확진사례가 보고되었다. 국내에서도 COVID-19가 유입된 이후 지역사회 유행이 지속되고 있으며 (Figure 1), 2만 5천여명의 사망자를 포함하여 약 1,977만건 이상의 확진사례가 보고되었다[2].

국내에서는 지난 2020년 COVID-19가 시작된 이후로 TTIQ 개입전략(Test-Trace-Isolate-Quarantine)을 통해 신속한 검사와 광범위한 추적조사를 수행함으로 감염자를 조기에 인지하고 감염경로를 차단하고자 하였다[3]. 그러나 2020년 말, COVID-19의 세번째 피크가 발생하고 이 시기부터 확진 당시 역학적 전파경로가 파악되지 않는 확진자의 비율이 증가하면서 지역사회에서 발견되지 않은 사례가 많아지게 되었다[4]. COVID-19 감염자 중 미확진 사례가 5~80%를 차지하고[5], 이들은 유증상자와 유사한 수준으로 바이러스를 배출하며[6,7], 의학적 치료를 필요로 하지는 않지만 지역사회 내 질병의 확산에 기여한다[8]. 따라서 세계보건기구에서는 지역사회 내 무증상 감염자를 포함한 정확한 COVID-19 유병규모를 파악하기 위해 인구집단 기반의 혈청역학조사를 권장하고 있다[9]. COVID-19의 혈청역학적 조사는 인구집단에서 자연감염 또는 백신접종으로 획득한 COVID-19에 대한 항체여부를 직접 측정함으로 인구집단의 COVID-19에 대한 면역수준을 모니터링하고 평가할 수 있게 된다[10-12].

2022년 11월 기준으로 전세계적으로 140개국에서 3,924개의 COVID-19 항체유병률 조사가 실시되고 있는 것으로 보고되고 있다[13]. 이러한 혈청역학조사 자료 중 일부를 기반으로 한 메타분석은 잘못된 표본틀과 방법론적 단점이 있는 연구를 제외하고 코로나19 추정치가 보고된 사례를 초과한다는 점을 강조하며, 본 조사는 COVID-19 대유행을 모니터링하고 향후 잠재적인 신종바이러스에 대비하기 위해 무작위 샘플링을 통한 높은 수준의 표준화된 혈청감시 수행의 중요성을 강조한다[14]. 영국에서는 팬데믹 초기 단계인 2020년 5월부터 23개월 동안 매달 15만명을 대상으로 무작위 표본추출 방식으로 설문조사를 실시했고, 실시간으로 지역사회 감염에 대한 평가와 지역사회 기반의 항체양성률에 대한 자료를 반복적으로 생성했다. 이 자료는 팬데믹 확산을 이해하고 효과적인 방역대응 전략을 수립하는데 기초가 된다[15]. 호주의 코로나19 혈청감시 네트워크(COVID-19 Serosurveillance Network)에서는 18세 이상 헌혈자의 잔여혈액을 사용하여 정기적으로 항체유병률을 산출하고 있다[16]. 미국에서는 2021년 9월부터 2022년 4월까지 임상시험을 위해 의료기관에서 채취된 혈액샘플을 이용하여 4주 단위로 N항체 유병률을 보고하였고[17], 이러한 편의샘플링의 한계를 극복하고자 2021-2022 NHANES의 조사에서 COVID-19 혈청데이터를 수집하기도 하였다[18]. 국내에서도 COVID-19 항체유병률 조사를 실시하였지만 대부분 편의추출을 통한 소규모 조사연구이었다. 또한 지역별, 연령별, 기타 사회인구학적 요인이 분석이 되지 않았고, 다른 목적의 조사체계를 활용한 잔여 검체 분석으로 대표성 있는 COVID-19 항체유병률을 산출하기에는 제한적이었다[4,12,19,20].

COVID-19 항체유병률은 조사모집단, 샘플링방법, 조사 시간 및 장소, 검체분석 방법 등에 따라 달라질 수 있다[11]. 따라서 국내의 정확한 COVID-19 감염규모를 추정하기 위해서는 표본의 대표성을 확보한 집단을 대상으로 하는 전국적인 조사가 필요하다. 국내에서는 지난 2008년부터 지역사회건강조사를 수행하고 있다. 이 조사는 질병관리청, 17개 광역시도와 258개 보건소, 34개 대학 간 컨소시엄을 기반으로 운영되고 있으며, 지역을 대표할 수 있는 표본추출 및 조사체계를 갖추고 있다[21,22]. 이에 한국 혈청 유병률 연구(K-SEROSMART)는 지역사회건강조사 체계를 활용하여 코로나19에 대한 전국단위 혈청 역학조사를 수행하는 것을 목표로 COVID-19 항체유병률을 산출하고자 하였다.

**MATERIALS AND METHODS**

*System for the community-based seroepidemiologic survey of COVID-19*

조사체계는 다음과 같다. 조사운영은 한국역학회가 질병관리청 국립보건연구원의 지원을 받아 총괄하였으며, 표본관리팀, 조사수행팀, 검체관리팀, 자료분석팀을 운영하였다. 표본관리팀에서는 표본의 대표성 확보를 위해 표본지점 추출 및 관리와 사후보정을 위한 가중치를 산출하였다. 조사수행팀에서는 가구방문 설문조사와 혈액 검체를 수집하였다. 검체관리팀에서는 혈액 검체의 운송 및 COVID-19 항체검사를 수행하였고, 자료분석팀에서는 설문과 항체검사 결과를 취합하여 데이터베이스를 구축하고 분석을 수행하였다. 또한 조사과정 모니터링 및 주요 의사결정을 위해 다학제연구위원회를 별도로 구성하였다. 특히, 조사수행팀은 지역사회건강조사의 컨소시엄을 활용하여[22] 지역사회 내 34개 대학, 258개 보건소, 132개 의료기관으로 구성하였다. 지역사회 내 대학에서는 각 지역의 조사대상자와 조사원, 채혈기관(보건소 또는 의료기관)을 연계하며, 조사과정 모니터링 및 조사원의 질 관리를 수행하였다. 보건소와 의료기관에서는 지역 내 채혈현황을 모니터링 하며, 채혈대상자 응대 및 채혈업무를 수행하였다 (Supplementary Material 1).

한편, 지역사회건강조사는 지역보건법(제4조)에 따라 2008년부터 질병관리청에 의해 시작되었다. 지역사회기반의 단면조사로 보건소 관할구역에 거주하는 만 19세 이상 지역주민을 대상으로 수행하고 있다. 매년 각 보건소별로 약 900명, 전국 약 23만명이 조사되고 있으며, 일대일 면접조사를 통해 지역단위의 건강행태, 만성질환 수준에 대한 건강통계자료를 생산하고 있다. 대표성 있는 표본 추출을 위해 동/읍면, 주택유형을 층화한 확률비례계통 추출법을 사용하여 표본지점을 선정하고 계통추출을 통해 표본가구를 추출하였다. 전국 258개 보건소, 34개 지역 대학이 컨소시엄을 이루고 있으며, 조사지표 및 수행체계를 표준화하였을 뿐 아니라 지역사회 내 네트워크를 구축함으로 지속가능한 지역사회 조사감시체계를 마련하였다. 생산된 통계자료는 지역보건의료계획 수립 및 보건사업의 성과를 평가하는 자료로 활용되고 있다.

*Sample selection*

본 조사를 통해 산출되는 COVID-19 항체유병률의 대표성을 확보하기 위해 표본규모는 10,000명으로 정하였다[23]. 조사 모집단은 조사시점에 각 표본지점의 주거용 주택(아파트, 일반주택)에 거주하고 있으며, 채혈이 가능한 만 5세 이상 지역주민으로 정의하였다. 본 조사는 가구단위 조사로 표본지점을 선정한 후 표본가구를 선정하였다. 표본 설계과정은 Figure 2와 같다. 먼저, 표본지점 선정을 위한 표본추출틀은 2021년 지역사회건강조사에서 기 추출된 표본지점 23,000개를 활용하였고 이 중 1,000개의 표본지점을 선정하였다. 2021년 지역사회건강조사에서 선정된 표본지점은 2021년 행정안전부의 주민등록인구자료와 국토교통부의 주택자료를 연계한 표본추출틀에서 통∙반/리 내 주택유형별 가구수를 기준으로 동∙읍/면 별로 할당된 표본지점 수만큼 확률비례계통추출법으로 선정되었다[21,22,24].

1,000개의 표본지점은 각 시∙군∙구별 인구수를 고려하여 비례배분법으로 할당하였다. 전국 258개 보건소가 모두 참여할 수 있게 보건소별로 2개의 표본지점을 우선 할당한 후, 남은 지점의 개수는 각 시∙군∙구별로 2022년 7월, 만 5세 이상 주민등록인구 수를 고려하여 분배하였다(Table 1). 표본추출틀에서 통∙반/리 내 가구 수를 기준으로 시∙군∙구별로 할당된 표본지점 수만큼 확률비례추출법으로 선정하였다. 이때, 참여자의 채혈장소 접근성을 고려하여 표본추출틀 내에서 도서산간 지역에 해당하는 지점은 제외하였다. 한편, 세종특별자치시의 경우 보건소가 1개소에 불과하지만 광역시도별 비교를 위한 표본의 대표성 확보를 위해 10개의 표본지점을 선정하였다. 표본지점별 표본가구선정은 계통추출법을 사용하였으며, 각 표본지점별 가구 수를 파악하여 5개의 본가구를 선정하였고 본가구별로 2개의 예비가구를 추가 선정하였다. 예비가구는 본가구에 해당하는 일련번호의 다음 번호를 가진 가구를 선정하였다.

*Survey process*

조사수행과정은 [Figure 3]과 같다. 조사대상으로 선정된 가구에 조사대상가구임을 알리는 안내문을 발송하였다. 이후 훈련된 조사원이 해당 가구를 방문하여 만 5세 이상 가구원을 대상으로 연구참여 동의서와 인체유래물 동의서를 획득한 후 설문조사를 수행하였다. 이때, 만 18세 이하 대상자인 경우 법정대리인의 동의를 획득하였다. 설문조사는 대면설문으로 진행하였고 건강상태, 질환력, COVID-19 확진 및 백신접종이력, 사회경제적 수준(학력, 소득, 세대유형) 등에 대해 조사하였다. 설문조사 후 조사원의 안내에 따라 채혈을 위한 방문 장소와 시간을 예약하였다. 채혈은 참여자의 거주지 관할 보건소에서 수행하였으며, 만 5세~18세는 각 보건소별로 연계된 의료기관에서 채혈하는 것을 권고하였다. 채혈프로토콜에 따라 성인은 4mL, 소아청소년은 2mL의 혈액을 채혈한 후, 해당 검체는 원심 분리(3,000rpm에서 10분)하여 냉장 보관하였다.

표본의 대표성을 확보하기 위해 표본지점 당 최소 3가구, 10명을 조사하였고 조사를 거부하거나조사부적합 가구(성인 미거주, 외국인 거주가구, 가구원 중 COVID-19 관련 증상이 있거나 격리중인 경우, 채혈을 위한 이동이 불가능한 경우 등)인 경우 각 본가구별로 배정된 예비가구로 조사가구를 대체하였다. 또한 각 지역별로 할당된 인원수만큼 조사가 되지 않은 경우 예비가구를 추가로 방문하여 조사를 수행하였다.

*Laboratory testing*

각 지역 보건소 및 의료기관에서 수집한 혈액 검체는 당일 검체분석 기관의 지역별 센터에 취합되어 매뉴얼에 따라 보관되다가 익일 오전에 중앙 검체 분석기관으로 취합되어 분석되었다. COVID-19 항체분석을 위해 전기화학발광 면역(Electrochemiluminescence immunoassay: ECLIA) 원리를 사용하는 Roche Cobas e801 분석기에서 제조업체의 프로토콜에 따라 Elecsys Anti-SARS-CoV-2(Roche, Mannheim, Germany) 시약을 이용하여 항체검사를 수행하였다. 이 검사의 임상적 민감도와 특이도는 각각 99.5%와 99.8%이었다[4,25]. 본 조사를 위한 전용장비와 백업장비를 지정하여 운영하였고, 숙련된 담당자가 검사를 수행하였다.

COVID-19 항체를 검출하기 위한 대표적 항원으로는 스파이크 단백질(spike protein)과 뉴클레오캡시드 단백질(nucleocapsid protein)을 사용한다. 스파이크 단백질은 바이러스가 인간세포와 연결될 수 있도록 하며, 뉴클레오캡시드 단백질은 코로나바이러스의 가장 풍부한 단백질로 바이러스 입자 조립과 방출에 관여한다고 알려져 있다[26]. S항체는 백신효과를 포함하여 효과적인 면역 반응의 지표로 쓰이고 N항체는 자연 감염의 바이오마커로 쓰인다[27].

COVID-19 항체 검사결과는 다음과 같다. S항체의 결과는 수치형태로 제공되었으며, cut-off index가 0.80U/mL이상일 때 S항체에 대해 양성이라고 판정하였고, 이는 자연감염 또는 백신접종으로 인한 항체가 형성되었음을 의미한다[28]. N항체의 결과는 반응 또는 비반응, cut-off index가 제시되었다. cut-off index가 1.0 이상일 때 N항체에 대하여 반응하고 이는 N항체에 대해 양성이라 판정하였고, 이는 이전에 자연감염으로 인한 항체가 형성되었음을 의미한다[28]. 이때 N항체에 대한 cut-off index가 1.0보다 높게 측정된 값의 정도가 검체에 존재하는 항체의 총량을 나타내는 것은 아니다.

*Statistical analysis*

COVID-19 항체유병률의 모수를 추정하기 위해 2022년 7월 주민등록인구 수를 기준으로 한 표본가중치를 적용한 SAS의 PROC SURVEYMEANS 프로시져를 사용하였다[22-24]. 표본가중치는 (1) 설계가중치, (2) 무응답보정가중치, (3) 사후층화보정가중치 3가지 요소를 모두 곱하여 산출하였다. (i) 설계가중치는 표본추출률의 역수로 표본추출률은 보건소 단위로 할당된 인원을 주민등록인구 수로 나눈 수이다. (ii) 무응답보정가중치는 무응답자와 응답자 간의 특성이 유사하다는 가정하에 보건소 단위별로 응답률에 역수를 취한 값을 산출하였다. (iii) 사후층화보정가중치는 항체유병률에 영향을 주는 성별과 연령대별 표본분포와 모집단의 분포와 동일하게 조정해주는 가중치로 각 시도 단위로 성별과 연령대별 주민등록인구 수를 설계가중치와 무응답보정가중치를 곱하여 합한 값으로 나눈 값이다.

항체유병률 값과 함께 95% 신뢰구간과 상대표준오차(Relative standard error: RSE)를 제시하였다. 상대표준오차는 표준오차를 해당 추정치로 나누어 백분율로 표시한 값으로, 가구조사인 경우 상대표준오차가 25%이하인 경우 추정 값에 대한 신뢰도가 높다고 해석한다[29].

또한 본 연구를 통해 산출된 N항체 유병률과 질병관리청에 의해 보고되고 있는 누적발생자 규모와의 차이를 미확진 감염율로 정의하였고[4], ~~N항체 유병 규모 중 미확진 감염율이 차지하는 분율을 미확진 감염분율로 정의하였다.~~ 이때, 성별, 연령별, 광역시도별 누적발생률과의 차이를 비교하기 위해 2021년 12월 인구수를 기준인구수로 한 연령표준화가중치를 적용하여 N항체 유병률을 추가로 산출하였다. COVID-19 누적발생률은 2020년 1월 20일부터 2022년 7월 31일까지 국내 COVID-19 확진환자 보고체계에 의해 보고된 누적확진자 수를 분자로, 2021년 12월 주민등록인구 수를 분모로 하여 산출하였다. 국내 COVID-19 확진환자 보고체계는 다음과 같다. 의료기관에서는 확진자(전문가용 신속항원검사 또는 응급용 선별검사 결과가 양성)를 최초로 인지한 후 24시간 이내 보건소로 신고하고 발생신고서를 COVID-19 정보관리시스템에 입력하게 된다. 보건소에서는 확진자(보건환경연구원 또는 검사수탁기관으로부터 양성결과 확인)를 최초로 인지한 후 24시간 이내 시도로 보고하고 발생신고서를 COVID-19 정보관리시스템에 입력하게 된다[30].

*Ethics statementl*

본 연구는 생명윤리법 제36조 및 시행규칙 33조, 생명윤리법 제2조 시행규칙 2조에 의거하여 공중보건상 긴급한 조치가 필요하고 국가나 지방자치단체가 공공복리나 서비스 프로그램을 검토평가하기 위해 직접 또는 위탁하여 수행하는 연구에 해당하여 질병관리청 기관생명윤리위원회의 심의면제를 승인받았다(2022-07-04-PE-A).

**Results**

가구방문조사는 2022년 8월 11일부터 8월 30일까지 수행하였고 채혈은 8월 12일부터 9월 5일까지 진행되었다. 5,041개 가구의 9,945명이 조사에 참여하였으며, 이 중 1,957가구의 4,008명은 본 가구에서 조사가 수행되었고 3,084가구의 5,938명은 예비가구에서 조사되었다. 이들의 특성별 S항체 유병률과 N항체 유병률은 Table 2와 같다.

S항체 유병률은 97.6% (95% CI: 97.2-97.9)이었다. 연령별로는 5-9세에서 81.5% (95% CI: 76.5-86.4)로 가장 낮았고 연령대가 증가하면서 S항체 유병률도 높아져 70대에서 99.4% (95% CI: 98.9-99.8)로 가장 높았다. 가구소득 및 세대유형별로 유의한 차이가 있었으며, 의사진단질병 개수가 많을수록 S항체 유병률도 높았다. 광역시도별로는 대구에서 93.6% (95% CI: 90.8-96.3)로 가장 낮았고 세종에서 99.5% (95% CI: 98.4-100.0)로 가장 높았으며, 광역시도별 S항체 유병률은 통계적으로 유의한 차이가 있었다[Figure 4A].

N항체 유병률은 57.1% (95% CI: 56.0-58.2)로 남성 56.4% (95% CI: 54.7-58.1), 여성 57.9% (95% CI: 56.4-59.3)로 성별에 따른 유의한 차이는 없었다. 연령대별로는 5-9세에서 82.1% (95% CI: 77.3-87.0)로 가장 높았고 연령대가 증가하면서 N항체 유병률은 감소하여 80세 이상에서 31.2% (95% CI: 27.2-35.2)로 가장 낮았다. 교육수준과 가구소득수준이 높아질수록 N항체 유병률도 증가하였으며, 세대유형별로는 1인가구에서 44.6% (95% CI: 41.3-47.9)로 가장 낮았고 2세대 유형에서 60.8% (95% CI: 59.2-62.4)로 가장 높았다. 광역시도별로는 울산에서 45.9% (95% CI: 37.7-54.1)로 가장 낮았고 부산에서 63.2% (95% CI: 59.3-67.2)로 가장 높았으며, 광역시도별 N항체 유병률은 통계적으로 유의한 차이가 있었다[Figure 4B].

조사대상자의 미확진 감염분율은 33.9%로 남성은 36.6%, 여성은 30.5%이었다. 연령대별로는 50-59세, 60-69세, 70-79세 순으로 각각 47.9%, 45.0%, 40.5%로 높았고 80세 이상에서 14.1%로 가장 낮았다(Table 3). 광역시도별로는 울산에서 19.1%로 가장 낮았고 부산에서 45.0%로 가장 높았으며, 두 지역 간 미확진 감염분율은 2.4배의 차이가 있었다[Figure 4C].

**DISCUSSION**

본 연구는 초기 오미크론 변종 바이러스를 중심으로 COVID-19 사례가 급증한지 수개월 후인 2022년 8월 11일부터 2022년 9월 5일까지 만 5세 이상의 지역주민 9,945명을 대상으로 코로나19에 대한 혈청역학조사를 실시했다. K-SEROSMART는 지역사회건강조사(CHS) 체계를 기반으로 국내의 대표성 있는 COVID-19 항체유병률을 조사하기 위해 설계되었다. S항체 유병률은 97.6%로 국민 대다수가 S항체를 보유하고 있는 것으로 파악되었다. 성별, 지역별로는 큰 차이가 없었으나, 5-9세에서는 81.5%로 다른 연령대에 비해 S항체 유병률이 낮았다. 국내에서는 2022년 8월 현재, 12-17세의 소아청소년과 5-11세의 고위험군 소아청소년에 대해 기초접종을 권고[31]하고 있음에도 불구하고 5-11세의 기초접종률은 1.5%에 불과하여 해당 연령층에서의 낮은 백신접종률[32]이 이에 영향을 미친 것으로 추정된다. 또한 5-9세의 N항체 유병률은 82.1%로 S항체 유병률과 비슷한 수준으로 해당 연령층에서 생성된 S항체는 자연 감염에 의한 것으로 추정된다.

N항체 유병률은 57.1%이었다. 성별에 따라서는 큰 차이가 없었지만 연령이 증가하면서 N항체유병률이 낮아지면서, 80세 이상에서는 약 70%가 미감염자로 추정되었다. 다른 연구에 따르면, 호주에서 2022년 8월 23일에서 9월 2일까지 수집된 5,005개의 혈액샘플을 통해 추정된 N항체는 65.2%이었고[16], 영국에서 2022년 6월 29일부터 7월 19일까지 수집된 13,787개의 혈액샘플을 통해 추정된 N항체 유병률은 73.4%이었다[33]. 이것은 우리나라가 COVID-19 확산방지에 성공했다고 해석될 수도 있고, 반면에 COVID-19 확산의 여지가 있다고 해석될 수도 있다[34]. 우리나라는 COVID-19 발생초기부터 TTIQ전략과 더불어 다양한 기관과의 긴밀하고 신속한 대응체계를 구축하였고, 마스크 착용과 사회적 거리두기, 백신접종 등 감염병 예방에 대한 국민들의 적극적인 협조가 있었다. 이로 인해 COVID-19 감염규모가 다른 나라에 비해 적었다고 할 수 있다. 특히, COVID-19 백신접종이 COVID-19 감염률, 중증도, 입원 및 사망률을 감소시킨다는 증거들이 뒷받침되고 있는데[35], 우리나라는 50세 이상 고령층을 COVID-19 백신 우선접종 대상으로 선정하였다. 그 결과 2022년 7월을 기준으로 50세 이상 연령층에서의 기초접종률은 96.7%, 3차 접종률은 87.0%로 매우 높은 수준이었고 연령층이 높아질수록 백신접종률은 높았다[36]. 본 조사 결과에 의하면 연령층이 높아질수록 N항체 양성률이 낮아지고 있어 백신접종의 효과가 이를 반영한 것으로 추정할 수 있다. 그러나 다른 한편으로는, 고령의 연령층이나 동반질환을 가지고 있는 경우 N항체 유병률이 낮다는 것은 이들은 여전히 자연감염의 위험이 있고 발병 시 고위험 환자군으로 전환될 수 있는 집단이 될 수 있으므로 지속적인 감시와 대응을 위한 논의가 필요할 것이다.

한편, 국내 COVID-19 미확진 감염분율은 33.9%이었다. 이는 지난 2022년 4월에 국민건강영양조사 참여자를 대상으로 한 잔혈검사에서 보고된 미확진 감염분율 18.3%[37]이었던 것에 비해 크게 증가하였다. 이는 오미크론 대유행에 따라 미진단감염자도 함께 증가한 것으로 보인다. 반면에, 미국의 2021-2022 NHANES조사를 통해 보고된 18세 이상 성인의 미확진 감염분율인 43.7%[18]에 비해서는 낮은 수준이다. 이는 앞서 기술한 대로 COVID-19 발생초기에 TTIQ정책에 따라 환자의 접촉자를 광범위하게 추적 관리한 영향이라고 볼 수 있다[3]. 그러나 2022년 초 오미크론 서지(surge) 이후에는 3T 정책이 완화되면서 지역 내 숨어있는 COVID-19 미확진 감염자의 비중이 더욱 증가했을 수 있다. COVID-19 미확진 감염분율은 지역사회 내 COVID-19 감염확산의 정도를 평가하는 중요한 척도이다[38-40]. 본 조사결과, 남성이나 50대, 60대에서 COVID-19 미확진 감염분율이 높았고 광역시도별로도 약 2.4배의 차이가 있었다. COVID-19 팬데믹의 피해는 개인의 사회경제적인 특성에 따라 불균형하게 나타났고, 이에 따라 감염병 대응역량과 정책에 대한 순응도에 차이가 있다는 연구결과들이 보고되고 있다[41-42]. COVID-19 미확진 감염분율의 차이에 영향을 미칠 수 있는 직업군, 고용형태와 같은 사회경제적 요인과 각 지역별 COVID-19 대응정책 등에 대한 추가조사 및 심층분석을 통해 사회경제적 특성을 고려하는 정책적 보완책이 마련될 필요가 있다.

본 조사는 기존에 구축되어 있던 지역 보건소와 대학과의 컨소시엄[21]을 이용하여 민간의료기관과 협력체계 구축함으로써 체계적이며 신속하고 비용효과적으로 COVID-19 항체유병률 조사를 수행할 수 있었다. 이러한 시도는 향후 기존 대상자들을 추적하여 COVID-19 항체의 변동양상을 파악하고, 지역사회의 COVID-19 감염확산과 대응을 지속적으로 모니터링하며, 나아가 신종감염병 감시체계로도 활용될 수 있는 기반이 될 것이다.

본 연구의 한계점으로는 첫째, 전체 또는 일부 집단에서 COVID-19 항체유병률이 과소추정되었을 수 있다. 본 조사는 지역사회 기반 단면조사로 COVID-19로 인한 입원이나 사망이 많았던 고령층은 조사대상에 포함되지 않아 이에 대한 생존편향이 있을 수 있다. 또한 자연감염에 의하더라도 노출된 병원체의 양, 개개인의 질병에 대한 감수성, 예방접종 및 자연감염의 시기, 기저질환, 돌파감염 여부 등에 따라 항체형성이 되지 않았을 수도 있고 시간경과에 따라 항체의 역가가 소실되었을 수도 있다[17,43-45]. 둘째, 채혈을 위해 지역 보건소나 의료기관을 방문이 어려운 소아청소년이나 젊은 연령층의 조사참여율이 낮았으나 조사참여여부에 따라 항체유병 여부에 차이가 없다는 가정하에 가중치를 부여함으로 모수를 추정하였다. 셋째, 본 연구에서는 질병관리청에 의해 보고되고 있는 COVID-19 누적발생자 정보를 이용하여 지역사회 내 미확진감염 규모를 추정하였다. 의료기관 또는 보건소를 방문한 환자에 대해서만 COVID-19에 대한 진단이 가능하였으므로 지역사회 내 미확진감염율은 과소추정되었을 수 있다.

그럼에도 불구하고 2022년 8월, 전국규모의 지역사회 기반 COVID-19 항체유병률 조사 결과, 우리나라 인구 대부분이 백신접종이나 자연감염으로 획득하는 S항체를 가지고 있었고, 자연감염자 3명 중 1명은 미확진감염자임을 확인하였다. 본 조사는 지역 보건소와 대학, 민간의료기관과의 협력을 통해 약 3주라는 짧은 기간 내에 체계적이고 비용효과적으로 대규모 인원을 조사함으로써, 우리나라를 대표하는 COVID-19 항체유병률을 산출한 국내 최초의 전국단위 조사이다. 이는 해당 지역의 지역사회 보건소, 대학, 뿐만 아니라 COVID-19의 감염확산과 대응을 지역에서 지속적으로 모니터링할 수 있는 감시체계의 기반을 마련한 것에 의의가 있다. 향후 대상자들에 대한 추적조사와 주기적인 조사와 더불어 질병관리청의 COVID-19 확진정보 및 백신접종이력, 건강보험공단의 이환정보 등 다양한 자료원과의 연계를 통해 지역사회 내 COVID-19 감염확산의 특성, 미확진 감염 및 항체 역가의 변동양상의 영향요인 등을 규명함으로써 COVID-19 방역정책수립의 근거자료로 활용될 수 있을 것이다.

**SUPPLEMENTARY MATERIALS**

Korean version is available at http://www.e-epih.org/.

Supplementary materials are available at http://www.e-epih.org/.

**CONFLICT OF INTEREST**

본 연구의 저자들은 이 연구에 대해 선언할 이해관계 상충이 없음.

**FUNDING**

본 연구는 질병관리청 국립보건연구원의 연구비 지원(2022-ER2603-00)으로 수행됨.

**REFERENCES**

| 1. World Health Organization. Rolling updates on coronavirus disease (COVID-19) updated 31 July 2020 [cited 2022 Dec 16]. Available from: https://www.who.int/emergencies/diseases/novel-coronavirus 2019/  events-as-they-happen. |
| --- |
| 2. CoronaBoard. COVID19 dashboard; 2022 Jul 31 [cited on 2022 Dec 21]. Available from: https://coronaboard.kr/ (Korean). |
| 3. Ashcroft P, Lehtinen S, Bonhoeffer S. Test-trace-isolate-quarantine (TTIQ) intervention strategies after symptomatic COVID-19 case identification. PLoS One 2022;17:e0263597. |
| 4. Yoon K, Kim J, Peck KR, Kim HS, Lee H, Hwang YS, et al. Seroprevalence of SARS-CoV-2 antibodies during the third wave of COVID-19 in the Seoul metropolitan area of Korea. Epidemiol Health 2022;44:e2022085. |
| 5. Yanes-Lane M, Winters N, Fregonese F, Bastos M, Perlman-Arrow S, Campbell JR, et al. Proportion of asymptomatic infection among COVID-19 positive persons and their transmission potential: a systematic review and meta-analysis. PLoS One 2020;15:e0241536. |
| 6. Zou L, Ruan F, Huang M, Liang L, Huang H, Hong, Z, et al. SARS-CoV-2 viral load in upper respiratory specimens of infected patients. N Engl J Med 2020;382:1177-1179. |
| 7. Lee S, Kim T, Lee E, Lee C, Kim H, Rhee H, et al. Clinical course and molecular viral shedding among asymptomatic and symptomatic patients with SARS-CoV-2 infection in a community treatment center in the Republic of Korea. JAMA Intern Med 2020;180:1447-1452. |
| 8. Gao Z, Xu Y, Sun C, Wang X, Guo Y, Qiu S, et al. A systematic review of asymptomatic infections with COVID-19. J Microbiol Immunol Infect. 2021;54:12-16. |
| 9. World Health Organization. A coordinated global research roadmap: 2019 novel coronavirus; 2020 Mar 12 [cited 2022 Dec 22]. Available from: https://www.who.int/publications/m/item/a-coordinated-global-research-roadmap. |
| 10. Centers for Disease Control and Prevention. Interim guidelines for COVID-19 antibody testing; 2022 Dec 16 [cited 2022 Dec 27]. Available from: https://www.cdc.gov/coronavirus/2019-ncov/hcp/testing/antibody-tests-guidelines.html?CDC_AA_refVal=https%3A%2F%2Fwww.cdc.gov%2Fcoronavirus%2F2019-ncov%2Flab%2Fresources%2Fantibody-tests-guidelines.html. |
| 11. Bobrovitz N, Arora RK, Cao C, Boucher E, Liu M, Donnici C, et al. Global seroprevalence of SARS-CoV-2 antibodies: a systematic review and meta-analysis. PLoS One 2021;16:e0252617. |
| 12. Nah EH, Cho S, Park H, Hwang I, Cho HI. Nationwide seroprevalence of antibodies to SARS-CoV-2 in asymptomatic population in South Korea: a cross-sectional study. BMJ Open 2021;11:e049837. |
| 13. Sero Tracker. Key insights; 2022 Nov [cited on 2022 Dec 21]. Available from: https://serotracker.com/en/Explore. |
| 14. Bergeri I, Whelan MG, Ware H, Subissi L, Nardone A, Lewis HC, et al. Global SARS-CoV-2 seropre-  valence from January 2020 to April 2022: a systematic review and meta-analysis of standardized population-based studies. PLoS Med 2022;19:e1004107. |
| 15. Elliott P, Ward H, Riley S. Population monitoring of SARS-CoV-2 infections via random sampling during the COVID-19 pandemic. Am J Public Health 2023;113:514-516. |
| 16. Australian COVID-19 Serosurveillance Network. Seroprevalence of SARS-CoV-2-specific antibodies among Australian blood donors: round 3 update; 2022 Nov 3 [cited 2022 Dec 21]. Available from: https://www.kirby.unsw.edu.au/sites/default/files/documents/COVID19-Blood-Donor-Report-Round3-Aug-Sep-2022%5B1%5D.pdf. |
| 17. Clarke KE, Jones JM, Deng Y, Nycz E, Lee A, Iachan R, et al. Seroprevalence of infection-induced SARS-CoV-2 antibodies - United States, September 2021-February 2022. MMWR Morb Mortal Wkly Rep 2022;71:606-608. |
| 18. Akinbami LJ, Kruszon-Moran D, Wang CY, Storandt RJ, Clark J, Riddles MK, et al. SARS-CoV-2 serology and self-reported infection among adults - National Health and Nutrition Examination Survey, United States, August 2021 - May 2022. MMWR Morb Mortal Wkly Rep 2022;71:1522-1525. |
| 19. Song SK, Lee DH, Nam JH, Kim KT, Do JS, Kang DW, et al. IgG seroprevalence of COVID-19 among individuals without a history of the coronavirus disease infection in Daegu, Korea. J Korean Med Sci 2020;35:e269. |
| 20. Noh JY, Seo YB, Yoon JG, Seong H, Hyun H, Lee J, et al. Seroprevalence of anti-SARS-CoV-2 antibodies among outpatients in southwestern Seoul, Korea. J Korean Med Sci 2020;35:e311. |
| 21. Kang YW, Ko YS, Kim YJ, Sung KM, Kim HJ, Choi HY, et al. Korea Community Health Survey data profiles. Osong Public Health Res Perspect 2015;6:211-217. |
| 22. Kim YT, Choi BY, Lee KO, Kim H, Chun JH, Kim SY, et al. Overview of Korean Community Health Survey. J Korean Med Assoc 2012;55:74-83(Korean). |
| 23. Kweon S, Kim Y, Jang MJ, Kim Y, Kim K, Choi S, et al. Data resource profile: the Korea National Health and Nutrition Examination survey (KNHANES). Int J Epidemiol 2014;43:69-77. |
| 24. Korea Disease Control and Prevention Agency. Korea Community Health at a glance 2020: Korea Community Health Survey (KCHS). Cheongju: Korea Disease Control and Prevention Agency; 2021(Korean). |
| 25. Muench P, Jochum S, Wenderoth V, Ofenloch-Haehnle B, Hombach M, Strobl M, et al. Development and validation of the Elecsys Anti-SARS-CoV-2 immunoassay as a highly specific tool for determining past exposure to SARS-CoV-2. J Clin Microbiol 2020;58:e01694-e016920. |
| 26. Gralinski LE, Menachery VD. Return of the coronavirus: 2019-nCoV. Viruses 2020;12:135. |
| 27. Lee N, Jeong S, Lee SK, Cho EJ, Hyun J, Park MJ, et al. Quantitative analysis of anti-N and anti-S antibody titers of SARS-CoV-2 infection after the third dose of COVID-19 vaccination. Vaccines(Basel) 2022;10:1143. |
| 28. Rashidzadeh H, Danafar H, Rahimi H, Mozafari F, Salehiabar M, Rahmati MA, et al. Nanotechnology against the novel coronavirus (severe acute respiratory syndrome coronavirus 2): diagnosis, treatment, therapy and future perspectives. Nanomedicine(Lond) 2021;16:497-516. |
| 29. Korean law information center. Guideline of sampling design and management; 2016 [cited 2022 Dec 20]. Available from: https://www.law.go.kr/LSW/admRulLsInfoP.do?admRulSeq=2100000042881(Korean). |
| 30. Korea Disease Control and Prevention Agency. COVID-19 for healthcare provider [cited 2022 Dec 20]. Available from: https://ncv.kdca.go.kr/hcp/page.do?mid=0202(Korean). |
| 31. Korea Disease Control and Prevention Agency. COVID-19 vaccination notice: 5-11years; 2022 [cited 2022 Dec 20]. Available from: https://ncv.kdca.go.kr/menu.es?mid=a10124030000(Korean). |
| 32. Korea Disease Control and Prevention Agency. Urging participation in COVID-19 vaccination for severe illness and mortality prevention in pediatric and adolescent populations; 2022 Aug 18 [cited 2022 Dec 20]. Available from: https://ncov.kdca.go.kr/tcmBoardView.do?brdId=3&brdGubun=31&dataGubun=&ncvCont  Seq=6826&contSeq=6826&board_id=312&gubun=BDJ(Korean). |
| 33. UK Health Security Agency. COVID-19 vaccine surveillance report week 35: 1 September 2022 [cited 2022 Dec 20]. Available from: https://assets.publishing.service.gov.uk/government/uploads/system/uploads/  attachment_data/file/1101870/vaccinesurveillance-report-week-35.pdf. |
| 34. Inbaraj LR, George CE, Chandrasingh S. Seroprevalence of COVID-19 infection in a rural district of South India: a population-based seroepidemiological study. PLoS One 2021;16:e0249247. |
| 35. Mohammed I, Nauman A, Paul P, Ganesan S, Chen KH, Jalil SM, et al. The efficacy and effectiveness of the COVID-19 vaccines in reducing infection, severity, hospitalization, and mortality: a systematic review.  Hum Vaccin Immunother 2022;18:2027160. |
| 36. Korea Disease Control and Prevention Agency. Urging COVID-19 vaccination for the prevention of severe illness and mortality, including older adults and other eligible individuals; 2022 Jul 28 [cited 2022 Dec 20]. Available from: https://ncov.kdca.go.kr/tcmBoardView.do?brdId=3&brdGubun-  =31&dataGubun=&ncvContSeq=6784&contSeq=6784&board_id=312&gubun=BDJ(Korean). |
| 37. Korea Disease Control and Prevention Agency. The results of COVID-19 antibody prevalence surveys indicate that the natural infection rate is higher than the cumulative incidence rate during the same period; 2022 Jun 14 [cited 2022 Dec 20]. Available from: https://www.kdca.go.kr/board/board.es?mid=a20501010000&bid=0015&act=view&list_no=719832#(Korean). |
| 38. Shakiba M, Nazemipour M, Heidarzadeh A, Mansournia MA. Prevalence of asymptomatic COVID-19 infection using a seroepidemiological survey. Epidemiol Infect 2020;148:e300. |
| 39. Byambasuren O, Cardona M, Bell K, Clark J, McLaws ML, Glasziou P. Estimating the extent of asymptomatic COVID-19 and its potential for community transmission: systematic review and meta-analysis. J Assoc Med Microbiol Infect Dis Can 2020;5:223-234. |
| 40. Nishiura H, Kobayashi T, Miyama T, Suzuki A, Jung SM, Hayashi K, et al. Estimation of the asymptomatic ratio of novel coronavirus infections (COVID-19). Int J Infect Dis 2020;94:154-155.  41. Abedi V, Olulana O, Avula V, Chaudhary D, Khan A, Shahjouei S, et al. Racial, economic, and health inequality and COVID-19 infection in the United States. J Racial Ethn Health Disparities 2021;8:732-742.  42. Garnier R, Benetka JR, Kraemer J, Bansal S. Socioeconomic disparities in social distancing during the COVID-19 pandemic in the United States: observational study. J Med Internet Res 2021;23:e24591. |
| 43. Krutikov M, Palmer T, Tut G, Fuller C, Azmi B, Giddings R, et al. Prevalence and duration of detectable SARS-CoV-2 nucleocapsid antibodies in staff and residents of long-term care facilities over the first year of the pandemic (VIVALDI study): prospective cohort study in England. Lancet Healthy Longev 2022;3:e13-e21. |
| 44. Nakagama Y, Komase Y, Kaku N, Nitahara Y, Tshibangu-Kabamba E, Tominaga T, et al. Detecting waning serological response with commercial immunoassays: 18-month longitudinal follow-up of anti-SARS-CoV-2 nucleocapsid antibodies. Microbiol Spectr 2022;10:e0098622. |
| 45. Schallier A, De Baets S, De Bruyne D, Dauwe K, Herpol M, Couck, P. Assay dependence of long-term kinetics of SARS-CoV-2 antibodies. Diagn Microbiol Infect Dis 2021;100:115403. |

**Table 1**. Distribution of PSUs by region in the Korea Seroprevalence Study of Monitoring of SARS-CoV-2 antibody Retention and Transmission

| Region | Community Health center (N) | Population | Priority allocation  (PSU) | Proration  (PSU) | Total  (PSU) |
| --- | --- | --- | --- | --- | --- |
| Seoul | 25 | 9,493,211 | 50 | 89 | 139 |
| Busan | 16 | 3,334,595 | 32 | 31 | 63 |
| Daegu | 8 | 2,374,120 | 16 | 22 | 38 |
| Incheon | 10 | 2,960,580 | 20 | 28 | 48 |
| Gwangju | 5 | 1,434,703 | 10 | 13 | 23 |
| Daejeon | 5 | 1,448,182 | 10 | 13 | 23 |
| Ulsan | 5 | 1,114,753 | 10 | 10 | 20 |
| Sejong | 1 | 380,889 | 2 | 8 | 10 |
| Gyeonggi | 48 | 13,589,362 | 96 | 126 | 222 |
| Gangwon | 18 | 1,539,178 | 36 | 14 | 50 |
| Chungbuk | 14 | 1,597,163 | 28 | 15 | 43 |
| Chungnam | 16 | 2,121,011 | 32 | 20 | 52 |
| Jeonbuk | 14 | 1,776,949 | 28 | 17 | 45 |
| Jeonnam | 22 | 1,825,534 | 44 | 17 | 61 |
| Gyeongbuk | 25 | 2,612,016 | 50 | 24 | 74 |
| Gyeongnam | 20 | 3,293,709 | 40 | 31 | 71 |
| Jeju | 6 | 678,491 | 12 | 6 | 18 |
| Total | 258 | 51,574,446 | 516 | 484 | 1,000 |

PSU, primary sampling unit; SARS-CoV-2; severe acute respiratory syndrome coronavirus 2.

**Table 2**. Prevalence of antibodies to SARS-CoV-2 based on socio-demographic characteristics of the Korea Seroprevalence Study of Monitoring of SARS-CoV-2 Antibody Retention and Transmission: August 12-September 5, 2022

|  | | N | Prevalence of anti-S | | | Prevalence of anti-N | | |
| --- | --- | --- | --- | --- | --- | --- | --- | --- |
|  |  |  | Weighted % (95% CI) | RSE | p-value^1)^ | Weighted % (95% CI) | RSE | p-value^1)^ |
| Overall | | 9,945 | 97.6(97.2-97.9) | 0.2 | - | 57.1(56.0-58.2) | 1.0 | - |
| Sex | Male | 4,474 | 97.2(96.6-97.7) | 0.3 | 0.0243 | 56.4(54.7-58.1) | 1.5 | 0.1873 |
|  | Female | 5,471 | 98.0(97.5-98.4) | 0.2 |  | 57.9(56.4-59.3) | 1.3 |  |
| Age-group | 5-9years | 297 | 81.5(76.5-86.4) | 3.1 | <0.0001 | 82.1(77.3-87.0) | 3.0 | <0.0001 |
|  | 10-19years | 757 | 92.9(90.9-94.8) | 1.1 |  | 69.3(65.8-72.8) | 2.6 |  |
|  | 20-29years | 887 | 98.8(98.1-99.6) | 0.4 |  | 60.7(57.1-64.2) | 3.0 |  |
|  | 30-39years | 934 | 98.4(97.5-99.3) | 0.5 |  | 61.5(58.1-64.9) | 2.8 |  |
|  | 40-49years | 1,337 | 98.6(97.9-99.3) | 0.4 |  | 59.7(56.8-62.6) | 2.5 |  |
|  | 50-59years | 1,660 | 99.2(98.7-99.7) | 0.2 |  | 54.5(51.9-57.2) | 2.5 |  |
|  | 60-69years | 1,994 | 99.2(98.8-99.7) | 0.2 |  | 50.4(47.9-52.8) | 2.5 |  |
|  | 70-79years | 1,450 | 99.4(98.9-99.8) | 0.2 |  | 43.0(40.2-45.8) | 3.3 |  |
|  | ≥80years | 629 | 97.3(95.8-98.8) | 0.8 |  | 31.2(27.2-35.2) | 6.5 |  |
| Education  (≥19years) | Primary school | 1,555 | 99.1(98.6-99.6) | 0.3 | 0.7211 | 40.1(37.4-42.9) | 3.4 | <0.0001 |
|  | Middle/High school | 3,702 | 98.9(98.5-99.3) | 0.2 |  | 54.4(52.5-56.2) | 1.7 |  |
|  | Postsecondary | 3,582 | 98.7(98.3-99.2) | 0.2 |  | 58.2(56.4-60.0) | 1.6 |  |
| Household income | <2,000 | 1,940 | 97.8(97.0-98.7) | 0.5 | 0.0075 | 43.1(40.5-45.7) | 3.1 | <0.0001 |
|  | 2,000-3,999 | 2,391 | 98.3(97.7-98.9) | 0.3 |  | 56.7(54.4-59.0) | 2.1 |  |
|  | 4,000-5,999 | 1,369 | 96.4(95.3-97.5) | 0.6 |  | 59.0(56.1-62.0) | 2.5 |  |
|  | 6,000-7,999 | 1,565 | 96.7(95.6-97.7) | 0.5 |  | 57.4(54.7-60.2) | 2.5 |  |
|  | ≥8,000 | 1,288 | 97.9(97.0-98.8) | 0.5 |  | 64.7(61.9-67.6) | 2.3 |  |
| Generational household | Single person | 1,234 | 98.4(97.5-99.3) | 0.5 | <0.0001 | 44.6(41.3-47.9) | 3.8 | <0.0001 |
|  | 1generation | 2,802 | 99.3(99.0-99.7) | 0.2 |  | 52.3(50.1-54.4) | 2.1 |  |
|  | 2generation | 4,195 | 96.4(95.8-97.1) | 0.3 |  | 60.8(59.2-62.4) | 1.4 |  |
|  | 3generation | 526 | 97.6(96.0-99.2) | 0.9 |  | 58.4(53.6-63.2) | 4.2 |  |
| Region | Seoul | 1,399 | 98.5(97.8-99.2) | 0.3 | <0.0001 | 61.4(58.7-64.2) | 2.3 | 0.0001 |
|  | Busan | 636 | 98.5(97.6-99.4) | 0.5 |  | 63.2(59.3-67.2) | 3.2 |  |
|  | Daegu | 382 | 93.6(90.8-96.3) | 1.5 |  | 53.5(48.2-58.8) | 5.1 |  |
|  | Incheon | 485 | 95.9(94.1-97.8) | 1.0 |  | 51.3(46.0-56.6) | 5.3 |  |
|  | Gwangju | 228 | 96.7(94.2-99.3) | 1.3 |  | 55.0(47.8-62.2) | 6.7 |  |
|  | Daejeon | 230 | 96.3(93.7-98.9) | 1.4 |  | 58.0(50.9-65.1) | 6.3 |  |
|  | Ulsan | 192 | 94.7(91.0-98.3) | 2.0 |  | 45.9(37.7-54.1) | 9.1 |  |
|  | Sejong | 100 | 99.5(98.4-100.0) | 0.5 |  | 57.3(47.4-67.1) | 8.8 |  |
|  | Gyeonggi | 2,173 | 98.2(97.6-98.8) | 0.3 |  | 57.7(55.5-59.9) | 2.0 |  |
|  | Gangwon | 495 | 98.4(97.1-99.8) | 0.7 |  | 57.9(53.3-62.6) | 4.1 |  |
|  | Chungbuk | 431 | 96.5(94.4-98.6) | 1.1 |  | 56.0(50.4-61.6) | 5.1 |  |
|  | Chungnam | 513 | 97.7(96.0-99.4) | 0.9 |  | 54.4(49.3-59.5) | 4.8 |  |
|  | Jeonbuk | 440 | 98.7(97.3-100.0) | 0.7 |  | 52.7(47.0-58.5) | 5.5 |  |
|  | Jeonnam | 611 | 98.1(96.7-99.4) | 0.7 |  | 52.1(47.8-56.4) | 4.2 |  |
|  | Gyeongbuk | 738 | 96.5(94.8-98.2) | 0.9 |  | 55.9(51.8-59.9) | 3.7 |  |
|  | Gyeongnam | 710 | 97.7(96.2-99.1) | 0.8 |  | 55.7(51.4-60.1) | 4.0 |  |
|  | Jeju | 182 | 97.7(94.4-100.0) | 1.7 |  | 60.7(53.1-68.4) | 6.4 |  |
| Seroprevalence was estimated with sampling weights using the population of registered residents in July 2022. SARS-CoV-2, severe acute respiratory syndrome coronavirus 2; CI, confidence interval; RSE, relative standard error; anti-S, antibodies to spike proteins; anti-N, antibodies to nucleocapsid proteins; KRW, Korean won.  1: From the Rao-Scott chi-square test. | | | | | | | | |

**Table 3**. Proportion of unreported infections by sex, age group, and region of the Korea Seroprevalence Study of Monitoring of SARS-COV-2 Antibody Retention and Transmission: August 12-September 5, 2022

| Characteristics | | Seroprevalence age-standardized rate of anti-N^1)^ (A) | Reported cumulative rate by KCDC with confirmed COVID-19^2)^ (B) | Proportion of unidentified infection  ((A-B)/A) |
| --- | --- | --- | --- | --- |
| Overall | | 57.2 | 37.8 | 33.9 |
| Sex | Male | 56.5 | 35.8 | 36.6 |
|  | Female | 58.0 | 40.3 | 30.5 |
| Age-group | 5-9years | 82.2 | 66.6 | 19.0 |
|  | 10-19years | 69.5 | 55.3 | 20.4 |
|  | 20-29years | 60.3 | 44.2 | 26.7 |
|  | 30-39years | 61.7 | 43.4 | 29.7 |
|  | 40-49years | 59.7 | 37.3 | 37.5 |
|  | 50-59years | 54.5 | 28.4 | 47.9 |
|  | 60-69years | 50.4 | 27.7 | 45.0 |
|  | 70-79years | 42.7 | 25.4 | 40.5 |
|  | ≥80years | 30.9 | 26.9 | 12.9 |
| Region | Seoul | 61.6 | 41.5 | 32.6 |
|  | Busan | 63.6 | 35.0 | 45.0 |
|  | Daegu | 53.5 | 33.9 | 36.6 |
|  | Incheon | 51.1 | 38.7 | 24.3 |
|  | Gwangju | 54.6 | 38.9 | 28.8 |
|  | Daejeon | 57.9 | 37.8 | 34.7 |
|  | Ulsan | 46.1 | 37.3 | 19.1 |
|  | Sejong | 53.7 | 40.2 | 25.1 |
|  | Gyeonggi | 56.9 | 39.1 | 31.3 |
|  | Gangwon | 59.6 | 36.3 | 39.1 |
|  | Chungbuk | 56.7 | 37.2 | 34.4 |
|  | Chungnam | 54.8 | 36.2 | 33.9 |
|  | Jeonbuk | 53.9 | 35.8 | 33.6 |
|  | Jeonnam | 54.0 | 34.3 | 36.5 |
|  | Gyeongbuk | 57.5 | 32.0 | 44.3 |
|  | Gyeongnam | 56.1 | 35.0 | 37.6 |
|  | Jeju | 60.2 | 39.2 | 34.9 |
| Values are presented as %.  KCDC, Korea Centers for Disease Control and Prevention; COVID-19, coronavirus disease 2019; Anti-N, antibodies to nucleocapsid proteins; SARS-COV-2, severe acute respiratory syndrome coronavirus 2.  1 Estimated with age-standardized weights using the population of registered residents in December 2021.  2 The cumulative incidence rate was calculated by using the cumulative number of people confirmed by KCDC to have COVID-19 through July 31, 2022 as the numerator and the registered resident population in December 2021 as the denominator. | | | | |
